# Graphene-Based Electrochemical Sensors for the Determination of Pharmaceutical- and Agricultural-Based Emerging Contaminants in Water

**DOI:** 10.1021/acs.analchem.5c07462

**Published:** 2026-04-30

**Authors:** Drochss Pettry Valencia, Gloria Crespo, Leonardo Muñoz-Rugeles, Gustavo Lara-Cruz, Andres Jaramillo-Botero

**Affiliations:** † Escuela de Química, Universidad Industrial de Santander, Carrera 27, Calle 9 Ciudad Universitaria, Bucaramanga, Santander AA 678, Colombia; ‡ iOMICAS Research Institute, 6469 Pontificia Universidad Javeriana, Calle 17 # 121B-155, Cali, Valle Del Cauca 760031, Colombia; § Chemistry and Chemical Engineering, California Institute of Technology, Pasadena, California 91125, United States

## Abstract

Emerging contaminants (ECs), such as pharmaceuticals and agrochemicals, persist in aquatic systems and are often underestimated by targeted chromatographic methods that quantify only compounds with available analytical standards. Here, we present a portable electrochemical platform based on laser-induced graphene (LIG) and screen-printed graphene (SPG) electrodes, which couples hydrogen peroxide–assisted redox mediation with chronoamperometric detection to quantify the cumulative burden of oxidizable organic contaminants in water. Using amoxicillin (AMX) as a model compound, the system achieved sub-ppb sensitivity (LOD = 0.085 ppb) with excellent linearity (*r* ^2^ > 0.99) across a wide dynamic range and strong agreement with HPLC-MS analysis. Under optimized electro-Fenton (EF) conditions, additional ECs—including diclofenac, ibuprofen, glyphosate, and estradiol—showed comparable responses, demonstrating broad applicability independent of molecular structure. Mass spectrometry confirmed peroxide-driven radical transformation of AMX, revealing hydroxylated derivatives and β-lactam ring-opened intermediates consistent with reactive oxygen species (ROS) chemistry. Unlike targeted chromatographic approaches, the electrochemical signal reflects the integrated pool of peroxide-reactive species, including transformation products not directly quantified by conventional methods. Application to real wastewater samples showed that the EF-assisted electrochemical response captures a broader oxidizable organic load than targeted HPLC-MS/MS. These findings establish peroxide-assisted graphene electrodes as a sensitive, low-cost, and field-deployable platform for rapid screening of total oxidizable contaminant burden in complex water matrices.

## Graphical abstract

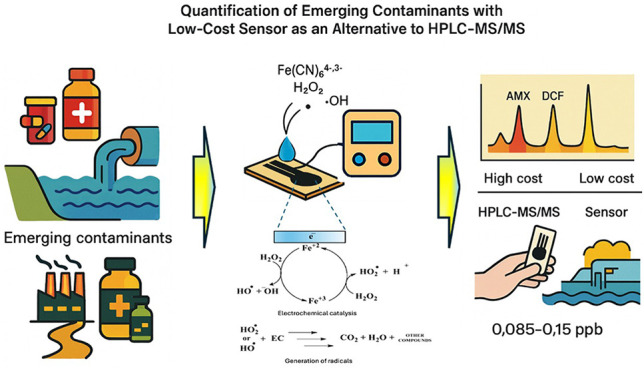

## 1 Introduction

Emerging contaminants (ECs), including pharmaceuticals and agrochemicals, are increasingly detected in surface waters and wastewater effluents at trace concentrations, raising concerns due to their persistence, biological activity, and potential cumulative effects on ecosystems and human health.^1–3^ Current monitoring strategies rely primarily on chromatographic techniques coupled with mass spectrometry, which provide excellent selectivity but remain costly, infrastructure-intensive, and inherently limited to compounds supported by analytical standards and spectral libraries.^3–5^ As a result, treatment efficiency and environmental burden are often underestimated when transformation products and unknown intermediates dominate the matrix.

Electrochemical sensors have therefore emerged as attractive complementary tools for rapid, low-cost, and field-deployable assessment of ECs in complex aqueous systems.^1,6,7^ In particular, carbon-based electrodes and graphene-derived materials offer favorable electron-transfer kinetics, chemical robustness, and tunable surface properties that can be exploited for signal amplification and enhanced sensitivity.^8^ Recent advances highlight the growing role of electrochemical platforms not only for target-specific detection but also for integrative measurements that respond to the global redox activity of complex contaminant mixtures.^9^

In this work, we report an electrochemical sensing platform based on laser-induced graphene (LIG) and screen-printed graphene (SPG) electrodes coupled with an electro-Fenton (EF) process and chronoamperometric readout for the determination of emerging contaminants in water. Amoxicillin (AMX) was selected as a representative pharmaceutical model due to its widespread use and frequent environmental occurrence,^10–12^ while additional pharmaceutical and agriculturally relevant ECs were evaluated to demonstrate the broader applicability of the method. Unlike targeted chromatographic approaches, the proposed strategy quantifies the total pool of electroactive and reactive oxygen species (ROS)-responsive contaminants, providing an integrated measure of oxidative contaminant load that is particularly relevant for assessing treatment performance and real-water matrices.

## 2 Materials and Methods

### 2.1 Reagents and Solutions

All reagents used were of analytical grade. AMX trihydrate (C_16_H_19_N_3_O_5_S·3H_2_O, 98.0%), ibuprofen sodium salt (≥98%), and diclofenac sodium salt were provided by Sigma-Aldrich. The agricultural herbicide glyphosate (active roundup) was provided by Bayer. A phosphate-buffered saline tablet (PBS 1x final concentrations are 137 mmol L^–1^ sodium chloride, 10 mmol L^–1^ phosphate, 2.7 mmol L^–1^ potassium chloride; pH is 7.40, Sigma-Aldrich). H_2_O_2_ 30% in aqueous solution was provided by Sigma-Aldrich, and potassium ferri/ferrocyanide K_3_[Fe­(CN)_6_/K_4_[Fe­(CN)_6_] (>99.0%, Sigma-Aldrich). Dimethylformamide (DMF, 99.8%) was provided by Merck. Buffer solutions, pH 4.01, 7.01, and 10.1, were all provided by Hanna traceable to NIST. The methanol hypergrade for LC-MS and formic acid for LC-MS used for AMX analysis in liquid chromatography were of ultrahigh purity UHPLC grade and were acquired from Merck. All solutions were prepared with deionized water with a resistivity of 18 MΩ·cm obtained in an ultrapure water system with ultrafiltration membrane and UV-photo-oxidation, type Smart2Pure 6 UV/UF.

A stock solution of 20 mmol L^–1^ of K_3_[Fe­(CN)_6_/K_4_[Fe­(CN)_6_] was prepared in 0.10 mmol L^–1^ PBS buffer at pH 7.40. From this stock solution, all solutions were prepared at 2.0 mmol L^–1^ of K_3_[Fe­(CN)_6_/K_4_[Fe­(CN)_6_]. In view of the limited water solubility of amoxicillin (AMX), stock solutions were prepared in a mixture of dimethylformamide (DMF) pure water (1:2, v/v). Stock solutions of 2000, 1000, and 500 mg L^–1^ of AMX were prepared in 0.10 mmol L^–1^ PBS buffer at pH 7.40 and stored at 4 °C until further diluted with ultrapure water or PBS to achieve the desired working concentrations. For chromatographic analysis, AMX stock solutions were prepared in a mixture of methanol hypergrade (Merck) + H_2_O type I (90:10). These solutions were stored at −80 °C to prevent decomposition. In order to ensure analytical reproducibility, all stock solutions were prepared fresh daily to minimize concentration fluctuations and potential degradation, especially related to the decomposition of hydrogen peroxide (H_2_O_2_).

### 2.2 Instruments

All electrochemical measurements were carried out in an Emstata4S potentiostat (Palmsens) connected to a PC with PS Trace 5 software. The electrochemical cell used is based on screen-printed graphene (SPG) and laser-induced graphene (LIG). In both cases, the working electrode (WE) consists of a circle with a diameter of 4 mm, a counter electrode (CE) whose area is three times the area of WE and a reference electrode (RE) to which we applied a silver/silver chloride ink (Ag/AgCl). The WE and CE are made of the corresponding carbon allotrope, i.e., printed graphene paste for the SPG and porous graphene for the LIG. A vortex shaker (DLAB MX-S) was used to mix all the prepared solutions. To control the pH in the samples, a Hanna Edge pH meter was used.

### 2.3 Fabrication and Preparation of Electrodes

Before use, the LIG and SPG sensors were subjected to air plasma cleaning to change their surface from hydrophobic to hydrophilic. This process favors uniform contact of the sample with the sensor, improving its absorption on the graphene. The SPG electrode shown in Figure S.1.A, was treated for cleaning with a low-pressure air plasma at a constant air flow of 7 sccm and a power of 50% (1200 VA) for 5 min using a Henniker HPT-100 plasma generator for cleaning electrode surfaces. On the other hand, LIG electrodes (Figure S.1.B) were engraved from 125 μm PI films acquired from Shijiazhuang Dadao Packaging Materials Co. LTD, SDPMC. The PI film was cleaned with a cloth soaked in 70% ethanol and then evaporated at room temperature. It was then heated at 150 °C for 30 min to prevent possible deformation during the LIG electrode fabrication process. The PI was coupled to an adhesive substrate (130 μm thick vinyl) to facilitate sample management. LIG electrodes were produced using the Universal Laser Systems VLS2.30DT CO_2_ laser engraver at 12% power (3.6 W), 15% speed (cm s^–1^), an image density of 5 units, and in raster mode. The operation of the printer was precisely controlled using unit’s UCP software. After the LIG sensors were fabricated, cleaning was performed with the low-pressure plasma under the same conditions as the SPG electrodes, to modulate the hydrophilic properties and improve the homogeneity of the surface of the printed LIG electrode for electrochemical applications. Further details on the choice of lasing parameters can be found in de la Roche et al.^13^

### 2.4 Electrochemical Measurements

The electrochemical characterization of the carbon surfaces was carried out using cyclic voltammetry (CV). First, a series of blank measurements were performed in a 2.00 mmol L^–1^ Fe­(CN)_6_^3–^/Fe­(CN)_6_^4–^ solution prepared in 0.10 mol L^–1^ phosphate-buffered saline (PBS) to ensure signal stability and reproducibility (usually three replicates). The cyclic scan was performed at a scan rate of 50 mV s^–1^ from 0.2 V to −0.2 V, followed by a change of scan direction and a sweep to 0.85 V and then back to 0.2 V, both for the ferricyanide solution and for a control solution without redox probe. The detection mechanism relies on ferri/ferrocyanide-mediated oxidation enhanced by H_2_O_2_, which behaves analogously to an electro-Fenton-like radical-generating environment but does not involve free Fe^2+^/Fe^3+^ chemistry.

Before starting the experimental measurements, this blank test protocol was repeated until a reproducible electrochemical response with a deviation of less than 2% was obtained for the LIG and SPG sensors (see Figure S.1, Supporting Information).

For the chronoamperometry (CA) measurements, solutions containing various concentrations of AMX in 0.10 mmol L^–1^ PBS buffer at pH 7.40 + Fe (CN)_6_^4–^ 2.00 mmol L^–1^ + 80 mmol L^–1^ H_2_O_2_ were measured from a potential of 0.00 V for 30 s to a final potential of 0.8 V for 50 s, using current data at different time points to generate curves. Current work *i*_(postpulse)_–*i*_(prepulse)_ vs AMX concentration in parts per million (ppm) and parts per billion (ppb). Calibration curves of AMX were performed by CA by adding increasing concentrations of the target compounds. Calibrations were performed in triplicate and all experiments in this study were performed at room temperature (23.0 ± 2.0 °C). To validate the electrochemical remediation with respect to the HPLC-MS technique, AMX was evaluated using different remediation times (200, 400, and 600 s).

### 2.5 Surface and Morphological Characterization

Surface morphology and structural characterization of the working electrodes (LIG and SPG) were evaluated before and after the EF process. Field emission scanning electron microscopy (FE-SEM) images were acquired using a Tescan Clara microscope equipped with an energy-dispersive X-ray (EDX) analysis system. Micrographs were obtained with a low-energy backscattered electron (LE-BSE) detector at magnifications of 1,000×, 4,000×, 10,000×, 30,000×, and 100,000×, operating at an accelerating voltage of 10 kV. Porosity and surface heterogeneity were estimated by digital image analysis using ImageJ software. For each electrode type (LIG and SPG), three independent images per magnification were analyzed to determine the surface roughness factor (RF). RF was calculated as the average of at least three measurements under different imaging configurations, both before and after EF operation.

### 2.6 Analytical Measurements

AMX was analyzed using a Shidmazu’s (Kyoto, Japan) HPLC-MS 9030 system equipped with a Shimadzu Nexcol C18 separation column (1.8 μm, 50 × 2.1 mm id). In order to develop the method for the analysis, method development was performed using ultrapure water with 0.1% (V/V) formic acid (eluent A) and methanol with 0.1% (V/V) formic acid (eluent B). Chromatographic separation was achieved with the following elution gradient: 90% A (0 min), 50% A (4 min), 5% A (17 min), 5% A (25 min), 90% A (25.1 min), and finally 90%A (30 min). The column temperature was maintained at 40 °C, and the mobile phase flow rate was 0.2 mL/min. Ultraviolet detection was performed between 190 and 800 nm using diode array detector (PDA). Prior to injection, the samples were prefiltered through a 0.22 μm cellulose nitrate membrane filter (Agilent Technologies) and 20 μL of each sample was injected into the HPLC-MS.

Mass spectrometric analysis was performed using an electrospray ionization source (ESI) operating in positive ion mode with a collision energy of 30 ± 10 eV. The analysis was performed in MS, MS/MS and Multiple Reaction Monitoring (MRM) modes, which allowed compound identification, monitoring of the most abundant precursor ions and precise quantification of AMX.

### 2.7 Density Functional Theory (DFT) Calculations

Density functional theory (DFT) calculations were performed to evaluate the feasibility of the main oxidation pathways potentially occurring under electro-Fenton (EF) conditions: single-electron transfer (SET), formal hydrogen transfer (FHT), and radical adduct formation (RAF). Formal one-electron reduction potentials (E°′, V vs Ag/AgCl sat.) were computed for the predominant acid–base microspecies of each EC. Geometry optimizations of reactants and radical products were carried out using the ωB97X-D^14^ density functional with the 6–311+G­(2d,p) Pople triple-ζ basis set. Solvent effects were modeled using the integral equation formalism of the polarizable continuum model (IEFPCM),^15^ employing the radii and nonelectrostatic terms of the SMD universal solvation model for water.^16^ All optimized structures were confirmed as true minima by frequency calculations showing no imaginary frequencies. SET calculations were performed using the Gaussian 16 software package.^17^

For AMX, additional mechanistic analysis of FHT and RAF pathways was conducted using condensed Fukui indices (*f* ^0^)^18^ derived from Hirshfeld population analysis.^19^ Because AMX presents multiple conformers and protonation states at different pH values, especially at pH 7.0, conformational sampling of relevant microspecies was performed using the Conformer–Rotamer Ensemble Sampling Tool (CREST, v2.11.2)^20^ with the GFN2-xTB semiempirical method^21^ and the Analytical Linearized Poisson–Boltzmann (ALPB)^22^ implicit solvation model for water. The lowest-energy conformers were subsequently reoptimized at the B3LYP-D4/def2-TZVP^23–25^ level with the SMD implicit solvent model. Frequency calculations confirmed local minima. Single-point energy calculations for Fukui index evaluation were carried out using the ωB97X^14^ functional with D3 dispersion corrections and the aug-cc-pVTZ basis set.^26^ All B3LYP-D4 and ωB97X-D3 calculations were performed with ORCA 6.1.0,^27^ employing the auxiliary basis set def2/J^28^ and default integration grid settings.

Detailed computational procedures, including oxidation potential corrections, Marcus theory treatment for SET kinetics, and full Fukui index analysis, are provided in the Supporting Information, Section S.1.

## 3 Results and Discussion

### 3.1 Electrocatalytic Oxidation of AMX Mediated by Fe­(CN)_6_^3–^/^4–^

The electrochemical behavior of the Fe­(CN)_6_^3–^/^4–^ redox probe and amoxicillin (AMX) was first evaluated by cyclic voltammetry (CV) under controlled experimental conditions.^29–38^ Potential scans were performed from the open-circuit potential (OCP) to 0.80 V, then reversed to −0.35 V, and finally returned to the initial potential at a scan rate of 50 mV s^–1^. As shown in Figure 1A, the Fe­(CN)_6_^3–^/^4–^ system exhibited a chemically and electrochemically reversible redox process, characterized by a well-defined anodic peak (*I*_o_) at 0.193 V and a cathodic peak (*I*_r_) at 0.01 V, with peak currents of approximately +29 μA and −30 μA, respectively. This behavior is consistent with outer-sphere electron-transfer mechanisms and has been widely reported for ferri/ferrocyanide at carbon-based electrodes.^29–33^ In contrast, AMX at 5.0 mmol L^–1^ (1820 ppm), dissolved in 0.10 mol L^–1^ PBS (pH 7.45), displayed only a weak and broad oxidation feature near 0.75 V (Figure 1A, black curve). This negligible faradaic response indicates that AMX is electrochemically inactive within the examined potential window at LIG electrodes. AMX oxidizes only above ∼1.0 V on carbon electrodes. Similar behavior has been documented for β-lactam antibiotics at glassy carbon, boron-doped diamond, and graphene-based electrodes, where direct electrooxidation is kinetically unfavorable due to high activation barriers and complex multielectron pathways.^39^

**Figure 1:**
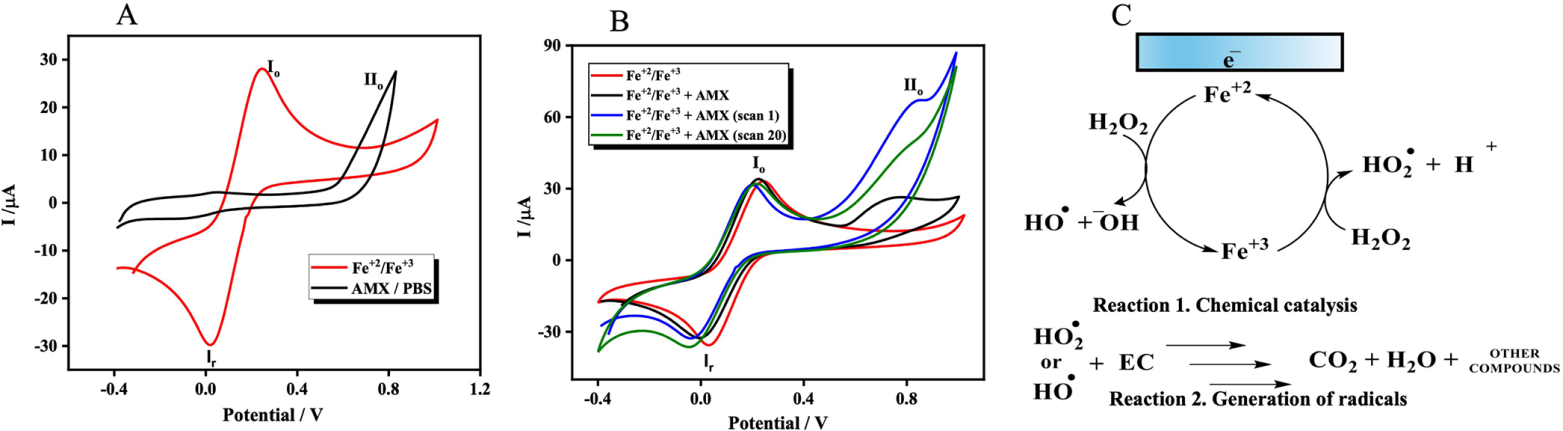
Electrochemical behavior of the catalyzed oxidation of AMX 5.00 mmol L^–1^ in the presence Fe^2+^/Fe^3+^ + H_2_O_2_ by CV at 50.0 mV/s in electrode SPG. A) The CV behavior of the Fe^2+^/Fe^3+^ system is shown in red, and the CV of AMX in PBS shown in black. B) CV at 50.0 mV/s in electrode SPG. CV Fe^2+^/Fe^3+^ in red, AMX 5.0 mmol L^–1^ Fe^2+^/Fe^3+^ 2.0 mmol L^–1^, in black the electrochemical oxidation of AMX 5.0 L^–1^ Fe^2+^/Fe^3+^ 2.0 mmol L^–1^, the blue scan corresponds to the oxidation of AMX in the presence Fe^2+^/Fe^3+^ H_2_O_2_ 80.0 mmol L^–1^, and green is as above but cycle 20. C) Proposed catalytic mechanism for the oxidation of AMX + Fe^2+^/Fe^3+^ system + H_2_O_2_.

Figure 1B shows the CV response of AMX^38^ in the presence of the Fe­(CN)_6_^3–^/^4–^ mediator, both with and without hydrogen peroxide. The reversible Fe^3+^/Fe^2+^ peaks (*I*_o_, *I*_r_) appear at similar potentials as inFigure [Fig fig1]A, confirming that the redox mediator remains electroactive in AMX-containing matrix. However, the addition of AMX produces a new anodic feature (*II*_o_) centered at approximately 0.80 V, indicating a mediated electrocatalytic oxidation pathway. The appearance of peak *II*_o_ is consistent with an electrochemical-chemical (EC’) catalytic mechanism, in which oxidized Fe­(CN)_6_^3–^ chemically oxidizes AMX in solution, followed by electrochemical regeneration of Fe­(CN)_6_^3–^ at the electrode surface.^40–43^

Upon addition of 80 mmol L^–1^ H_2_O_2_ (Figure 1B, blue and green scans), a pronounced increase in anodic current is observed at potentials above 0.78 V, along with the progressive decline of II_o_ after sequential cycles. This behavior is characteristic of electro-Fenton-like catalysis, in which Fe^3+^ reacts with H_2_O_2_ to produce highly reactive oxygen species (ROS), including •OH and HO_2_• radicals, which then oxidize AMX and other organic contaminants. These ROS-driven reactions decompose AMX into electrochemically silent products (CO_2_, H_2_O, and heteroatom-containing gases), while the Fe­(CN)_6_^3–^/^4–^ couple remains catalytically active.^2–5,44–48^ The stability of the *I*_o_ and *I*_r_ peaks across multiple cycles, contrasted with the consistent attenuation of *II*_o_, strongly supports the conclusion that AMX oxidation arises from a mediated electrocatalytic cycle rather than from direct electrode oxidation. This observation is fully aligned with iron-mediated oxidative pathways previously described for electrochemically catalyzed surface modifications.^40,41,49,50^

Figure 1C presents the proposed mechanistic pathway for the oxidation of emerging contaminants in the presence of hydrogen peroxide, mediated by the Fe­(CN)_6_^3–^/^4–^ redox couple at graphene-based electrodes. The Fe­(CN)_6_^3–^/^4–^ species undergo rapid, reversible electron transfer at the electrode surface, enabling a continuous redox cycle that promotes the electrochemical conversion of Fe^2+^ to Fe^3+^. In the presence of H_2_O_2_, the chemically generated Fe^3+^ initiates the formation of reactive oxygen species (ROS), •OH and •OOH radicals, as shown in eqs 1 and 2:

1 $${\mathrm{Fe}}^{3+}+H_{2}O_{2}\to {\mathrm{Fe}}^{2+}+\cdot \mathrm{OOH}+H^{+}$$

2 $${\mathrm{Fe}}^{2+}+H_{2}O_{2}\to {\mathrm{Fe}}^{3+}+\cdot \mathrm{OH}+{\mathrm{OH}}^{-}$$

These ROS species attack AMX, yielding fragmented intermediates that undergo further oxidation until full mineralization into CO_2_ and H_2_O. The Fe­(CN)_6_^3–^/^4–^ species is not consumed but is continuously regenerated electrochemically, allowing repeated catalytic cycles. This synergistic redox mediation and ROS generation significantly enhance the rate and extent of AMX degradation compared with direct electrochemical oxidation. Thus, the combined action of Fe­(CN)_6_^3–^/^4–^, H_2_O_2_, and the high surface reactivity of graphene electrodes not only accelerates AMX degradation but also establishes a robust mechanistic foundation for simultaneous detection and electrochemical remediation of ECs.

### 3.2 Parameter Optimization for Improved AMX Detection

The electrocatalytic detection of AMX was systematically optimized by evaluating the effects of pH, hydrogen peroxide concentration, applied potential, reaction time, and CA measurement windows on the mediated EF-like oxidation mechanism. AMX at 1800 ppm (5.0 mmol L^–1^) was studied in the presence of the Fe^2+^/Fe^3+^ redox couple and H_2_O_2_ using CV and double-pulse chronoamperometry (CA). Because environmental waters exhibit pH values ranging from mildly acidic to alkaline depending on treatment processes and effluent composition, the pH was varied from 4 to 10 to assess the robustness of the system under realistic environmental conditions.^44–48^ Figure 2A shows that the Fe^2+^/Fe^3+^ redox couple maintains its quasi-reversible electron-transfer behavior across the entire pH range, with peak current variations below 10%, confirming the electrochemical stability of the mediator. In contrast, the AMX oxidation signal shows strong pH dependence. No defined oxidation feature is observed at extreme pH values, consistent with AMX’s acid–base transitions and the coexistence of different protonation states that alter electron-transfer kinetics. At near-neutral pH (≈7.6), a distinct oxidation peak appears at ∼0.78 V, consistent with a diffusion-controlled EC’ process in line with classical Savéant models for mediated electrocatalysis.^42,43,51,52^ The highest AMX oxidation currents were observed at pH ∼7.0, particularly above 0.50 V, indicating that the electrocatalytic pathway involving Fe­(CN)_6_^3–^/^4–^ and H_2_O_2_ is most efficient under near-neutral conditions. Based on these results and to ensure compatibility with real wastewater matrices, pH 7.01 was selected as the working condition. To further enhance analytical performance, a double-pulse CA method was used (Figure 2B). The initial potential (*E*_*i*_ = 0.00 V) was held for 30 s to establish a baseline and ensure complete reduction of electroactive species at the electrode–solution interface. The potential was then stepped to *E*_f_ = 0.80 V, the value identified from CV as optimal for activating the Fe^3+^/Fe^2+^-mediated oxidation of AMX via EF-like chemistry. The CA transients clearly show the characteristic decay predicted by the Cottrell relation, which indicates diffusion-controlled mediator regeneration rather than direct AMX oxidation.

**Figure 2:**
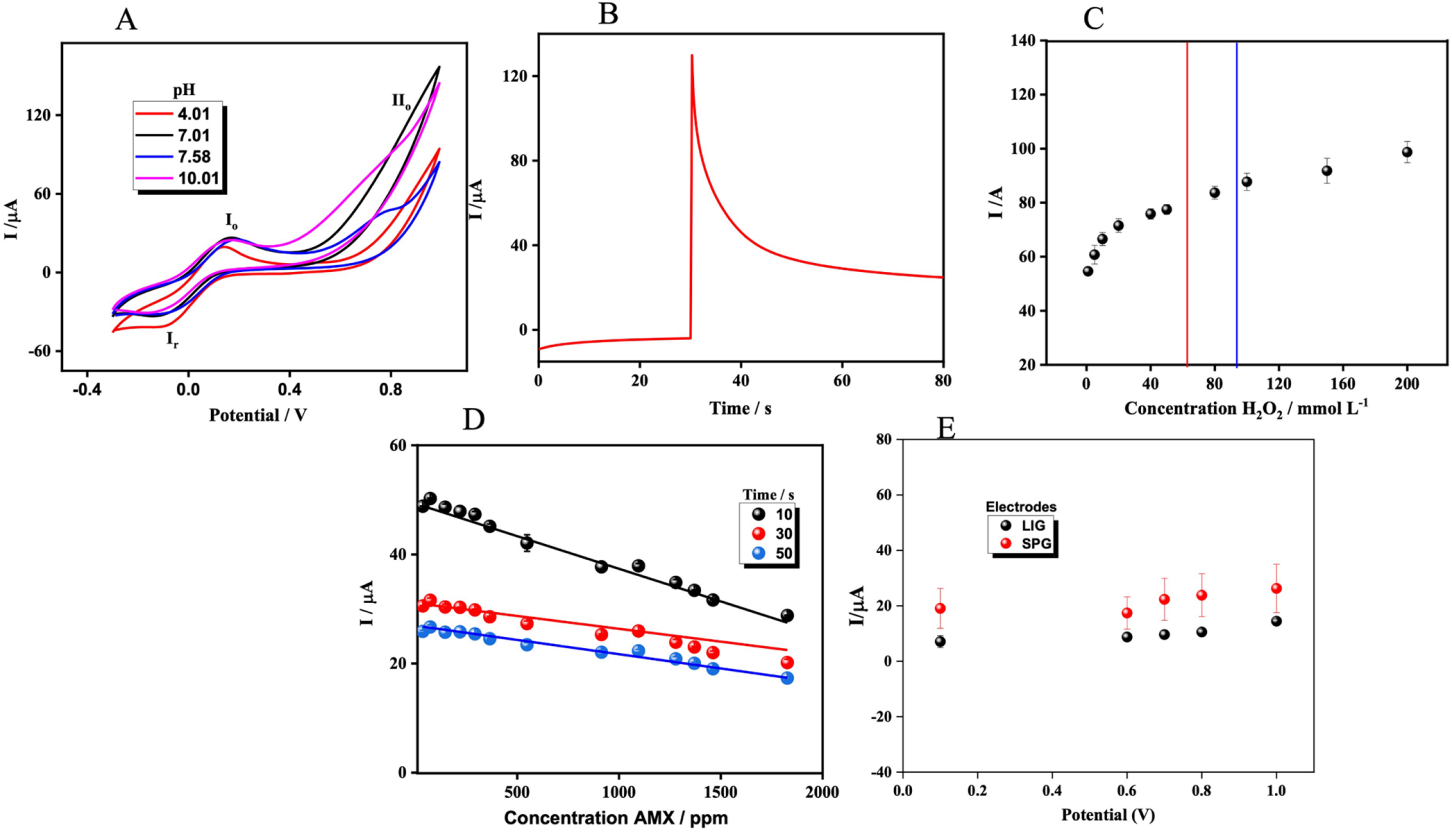
A) Effect of pH on the catalyzed electrochemical oxidation of AMX + Fe^2+^/Fe^3+^ + H_2_O_2_. B) Double-pulse potential chronoamperometry for the electrochemical oxidation of AMX, *E_i_* = 0.0 V for 30 s, *E*_f_ = 0.8 V for 50 s. C) Effect of H_2_O_2_ by CA, the regression equation was in black: *I* (μA) = −0.0119*x* + 49.321 (*R* ^2^ = 0.995), in red: *I* (μA) = −0.00471*x* + 31.080 (*R* ^2^ = 0.944), in blue: *I* (μA) = −0.0523*x*+ 26.945 (*R* ^2^ = 0.988). D) Optimization *I*(*t*)= 10 s. E) Study of the potential effect in solution 2.0 mmol L^–1^ Fe^2+^/Fe^3+^ by CA. All experiments were performed in 2 mmol K_4_[Fe­(CN)_6_] + 0.1 mol L^–1^ PBS buffer at a pH of 7.01 and a scan rate of 50 mV s^–1^.

Hydrogen peroxide (H_2_O_2_) concentration was optimized because H_2_O_2_ controls both the generation rate and the steady-state concentration of reactive oxygen species (ROS), particularly hydroxyl (•OH) and hydroperoxyl (HO_2_•) radicals, in electro-Fenton (EF) systems. As shown by the progressive increase in electrochemical current with rising peroxide concentration in Figure 2C, and as mechanistically proposed in Figure 1C, the measured response results from coupled redox processes involving peroxide activation and mediator cycling at the electrode interface. In classical Fenton chemistry, Fe^2+^ activates hydrogen peroxide to produce •OH radicals (eq 2); however, this homogeneous pathway is inherently limited by stoichiometric Fe^2+^ consumption and peroxide instability. In contrast, the electro-Fenton configuration overcomes these limitations by electrochemically regenerating the reduced species (eq 3) while simultaneously maintaining H_2_O_2_ availability through in situ oxygen reduction (eq 4).^1–3^

3 $$Fe^{3+}+e^{-}\to Fe^{2+}$$

4 $$O_{2}+2H^{+}+2e^{-}\to H_{2}O_{2}$$

The EF process maintains a dynamic redox equilibrium between Fe^2+^ and Fe^3+^ species, sustaining a continuous flux of ROS and enabling precise kinetic control over oxidation efficiency.^2–5^ This self-regenerating catalytic cycle increases the formation rates of •OH and HO_2_• radicals as a direct function of the initial H_2_O_2_ concentration; these radicals are the primary oxidizing agents responsible for pollutant degradation and mineralization.^6^ This kinetic dependence is directly reflected in the biphasic electrochemical response shown in Figure 2C. At low H_2_O_2_ concentrations (<30 mmol L^–1^), the anodic current rises sharply with increasing peroxide concentration, indicating a regime where ROS generation is limited by peroxide availability. In this region, the rate of Fe^3+^ reduction and subsequent chemical activation of H_2_O_2_ (eq 1) governs the overall oxidation kinetics, resulting in a proportional increase in radical flux and electrochemical signal.

Beyond this threshold, the change in current response becomes less pronounced, and further increases in hydrogen peroxide concentration do not significantly enhance the electrochemical signal. This behavior defines a second kinetic regime in which ROS generation reaches mechanistic saturation, and the oxidation rate becomes limited by the availability of oxidizable organic substrates and mass transport rather than peroxide concentration. Under these conditions, excess H_2_O_2_ promotes competitive scavenging pathways, leading to the formation of superoxide radical (O_2_•^–^) and hydroperoxide anion (HO_2_•^–^) (eqs 5–6). These species have significantly lower oxidation potentials and reaction rate constants toward organic contaminants compared to •OH, and therefore contribute less effectively to further oxidation and mineralization.^2–5^

5 $$HO_{2}\cdot ⇋{O_{2}}^{.-}+H^{+}$$

6 $$Fe^{2+}+HO_{2˙}\to Fe^{3+}+H{O_{2}}^{.-}$$

In addition to the dominant homogeneous pathway, multiple secondary homogeneous and heterogeneous reactions contribute to the overall oxidative performance of the system, with their relative importance determined by solution pH, electrode composition, and surface morphology.^1,7^ Under optimized electro-Fenton conditions, the Fe^2+^/Fe^3+^ redox couple efficiently activates H_2_O_2_ and sustains continuous ROS generation, thereby driving the oxidation of ECs. The reaction sequence summarized in eqs 4–8 captures the coupled electrochemical and chemical steps responsible for the progressive transformation of organic substrates (R-H), from initial radical attack to advanced oxidation and near-complete mineralization.^9,53^

7 $$\cdot OH+R-H\to R\cdot +H_{2}O$$

8 $$R\cdot +O_{2}\to RO_{2}\cdot \to CO_{2}+H_{2}O+NO_{x}+SO_{x}$$

Accordingly, the biphasic behavior observed in Figure 2C provides direct experimental validation of the mechanistic framework proposed in Figure 1C, establishing a quantitative link between peroxide-controlled ROS generation and the evolution of the electrochemical signal. Based on signal stability, catalytic efficiency, and reproducibility across complex matrices, an H_2_O_2_ concentration of 80 mmol L^–1^ was selected as the optimal operating condition for subsequent experiments.

To ensure accurate and reproducible quantification, key parameters of the CA protocol were also optimized. The reaction time (*R*_t_) was defined as the interval required to reach a stable catalytic regime in the CA transient. As shown in Figure 2D, the highest analytical sensitivity, expressed as the slope of the calibration curve, was obtained at *t* = 10 s, corresponding to a kinetically controlled EF regime. At longer delays (30 and 50 s), the slopes decrease markedly, indicating the onset of a depletion-controlled regime in which further oxidation does not result in proportional current changes. The consistently negative slope observed at all delays confirms that the measured current decay reflects AMX degradation mediated by ROS, rather than direct anodic oxidation at the electrode surface.

*E*_f_ was also systematically varied between 0.60 and 1.00 V (Figure 2E). For both LIG and SPG electrodes, increasing *E*_f_ resulted led to higher current responses due to accelerated mediator cycling and enhanced ROS generation under EF conditions. An operating potential of 0.80 V was selected as optimal, as it provides a suitable balance between sensitivity and signal-to-noise ratio while minimizing electrode overoxidation and parasitic reactions. Although SPG electrodes exhibited higher absolute currents across the investigated potential range, both platforms showed identical electrochemical behavior. CV showed comparable redox peak profiles (Figure 2A), and statistical comparison of the calibration slopes in Figure 2E revealed no significant differences (one-way ANOVA, *p* > 0.05), confirming that the EF-assisted sensing mechanism is preserved regardless of electrode architecture.

To explain the differences in current magnitude, SPG and LIG electrodes fabricated by distinct methods were directly compared. Both electrodes had the same geometric diameter (4 mm), and their electrochemically active surface areas, determined by CA and CV using 2.00 mmol L^–1^ Fe­(CN)_6_^3–^/Fe­(CN)_6_^4–^ + 0.10 mol L^–1^ PBS, were statistically indistinguishable when averaged over multiple electrodes, although SPG values were consistently slightly higher. SEM analysis performed before and after EF operation revealed markedly different morphological evolution. Initially, SPG electrodes exhibited a more homogeneous surface, reflected by a lower roughness factor (0.06483 ± 0.01298) compared to LIG (0.11204 ± 0.01834). After the electro-Fenton process, SPG roughness increased to 0.08297 (≈22% change), whereas LIG showed only a minor variation to 0.12008 ± 0.02337 (≈6% change).

This greater structural evolution in SPG electrodes explains their higher faradaic currents but also their increased background contribution and signal variability. In contrast, the minimal roughness variation observed for LIG indicates superior morphological stability under EF conditions, resulting in improved baseline stability, enhanced signal-to-noise ratios, and better reproducibility.^54–57^ Accordingly, despite the slightly higher raw currents obtained with SPG, LIG electrodes were selected as the preferred sensing platform due to their greater structural robustness, analytical precision, long-term stability under oxidative EF environments, and inherent fabrication and cost advantages.

### 3.3 Analytical Performance and Mechanistic Interpretation of the EF-Assisted AMX Response

Once the optimal electrochemical parameters were established (*E*_f_ = 0.80 V, CA reaction time = 10 s, 2.0 mmol L^–1^ Fe­(CN)_6_^3–^/^4–^ , pH 7.01, and 80 mmol L^–1^ H_2_O_2_), CA measurements were performed to evaluate the analytical performance of the EF-assisted sensor and to elucidate the mechanistic origin of the current–concentration relationship. Figure 3 summarizes the response over a wide AMX concentration range (0.01–2000 ppm), revealing two distinct linear regimes associated with different balances between ROS generation and consumption. All measurements were performed in triplicate (*n* = 3 per concentration).

**Figure 3:**
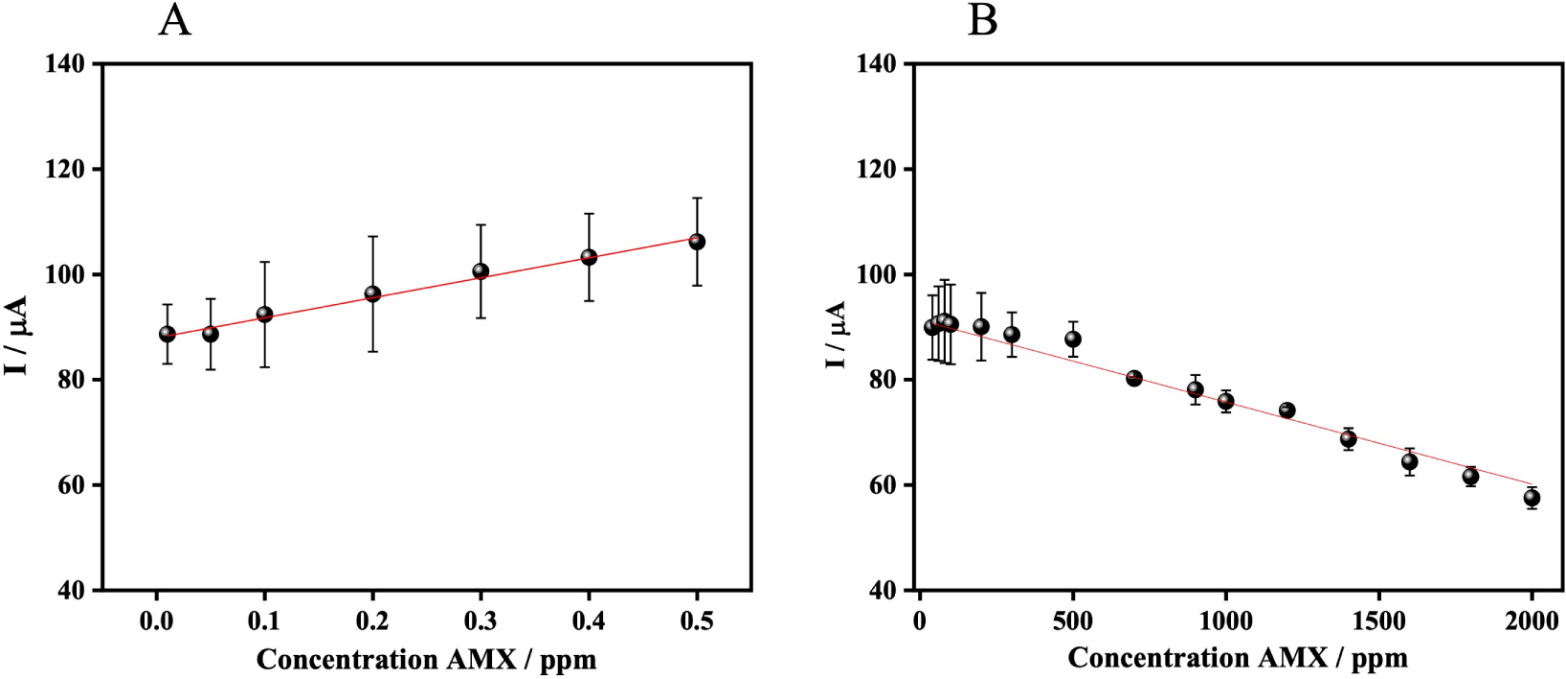
Calibration curves for AMX determination in LIG electrodes. A) Calibration curve in the concentration range (0.01–0.5 ppm). B) Calibration curve in the concentration range (1–2000 ppm). The curves were obtained by CA using the electrochemical oxidation methodology, each concentration point was measured in triplicate.

In the low-concentration regime (0.01–0.5 ppm; Figure 3A), the oxidation current increases proportionally with AMX concentration. Under these conditions, reactive oxygen species, mainly •OH and HO_2_• generated by the EF process, are present in relative excess compared to the analyte. In this regime, ROS consumption by AMX is limited, and the radical generation term dominates the system kinetics.^12,58^ The relationship between radical availability and oxidative response can be explained using the model proposed by Gualandi and Tonelli,^59^ which describes indirect radical quantification through fast-reacting probe molecules. According to this model, the concentration of the oxidized probe product is given by eq 9:

9 $$\left[Probe\;product\right]=\frac{\left[\cdot OH\right]\eta k_{p}\left[\mathrm{Probe}\right]}{k_{p}\left[\mathrm{Probe}\right]+k_{m}\left[\mathrm{Matrix}\right]}$$

Where [•OH] is the hydroxyl radical concentration, η is the effective reaction yield, *k_p_* is the rate constant for the probe–radical reaction, and *k_m_[Matrix]* accounts for competing radical scavenging by the surrounding medium. Because *k_m_[Matrix]* is generally unknown and often comparable to *k_p_[Probe]*, absolute quantification of •OH is not feasible. Nevertheless, a relative scale of radical generation can be established under fixed matrix conditions, providing a practical kinetic descriptor of EF efficiency.^59–61^

The overall ROS dynamics are further described by the simplified mass balance expressed in eq 10:^60,62–64^

10 $$\frac{d\left[ROS\right]}{dt}=k_{gen}\left(E\right)\left[Fe^{2+}\right]\left[H_{2}O_{2}\right]-k_{cons}\left[ROS\right]\left[R\right]$$

In the low-concentration regime, the consumption term *k*_cons_[ROS]­[R] remains small, and ROS generation controls the current response. Consequently, the measured current reflects an ROS-activated electrocatalytic regime, yielding the highest analytical sensitivity. The calibration in this region followed *I* (μA) = 37.99·C_AMX_ + 87.96 (*r* ^2^ = 0.984).

At higher AMX concentrations (>0.5 ppm; Figure 3B), the system transitions into a second linear regime characterized by a negative slope. In this region, the consumption term in eq 10 becomes dominant, as ROS are rapidly scavenged by AMX in bulk solution. The increased analyte concentration accelerates radical consumption, reducing the steady-state ROS concentration despite constant generation conditions. As a result, the faradaic current decreases with increasing AMX concentration, producing a saturation-like behavior characteristic of ROS-limited EF systems operating under high organic load. This response reflects a shift from a generation-controlled to a consumption-controlled kinetic regime, consistent with previous reports on EF-driven oxidation under diffusion and radical depletion constraints.^60,62–64^ The calibration in this region was *I* (μA) = −0.0156·C_AMX_ + 91.307 (*r* ^2^ = 0.968).

The coexistence of the two linear calibration regimes can be explained by the coupling between EF reaction kinetics and diffusion-controlled electrochemical behavior. Under CA conditions, the transient current follows the Cottrell relationship, which links charge transfer to analyte concentration and oxidative flux. Consequently, the measured electrochemical signal reflects the cumulative oxidative load associated with AMX and its early transformation products, rather than only the parent compound. This characteristic accounts for the systematic differences observed relative to targeted HPLC-MS/MS quantification and highlights the suitability of the EF-assisted electrochemical platform as a rapid, reproducible, and cost-effective tool for assessing emerging contaminants in complex water matrices.^65,66^

To confirm that the dual-regime behavior observed in Figure 3 is an intrinsic feature of the EF-mediated oxidation mechanism and not an artifact of a specific electrode architecture, the complete calibration protocol was repeated using SPG electrodes. Each AMX concentration was measured in triplicate (*n* = 3 per point) under identical EF-optimized conditions. As shown in Figure 4, SPG electrodes exhibited the same two well-defined linear regimes observed for LIG electrodes in both the low- and high-concentration ranges. Statistical comparison of the analytical sensitivities, performed by one-way ANOVA on the slopes of the corresponding calibration curves, showed that the slopes for each linear region were statistically indistinguishable between the LIG and SPG platforms (>98% similarity). These results confirm that the observed dual-regime response originates from the intrinsic kinetics of the EF-mediated oxidation process rather than from electrode-specific effects.

**Figure 4:**
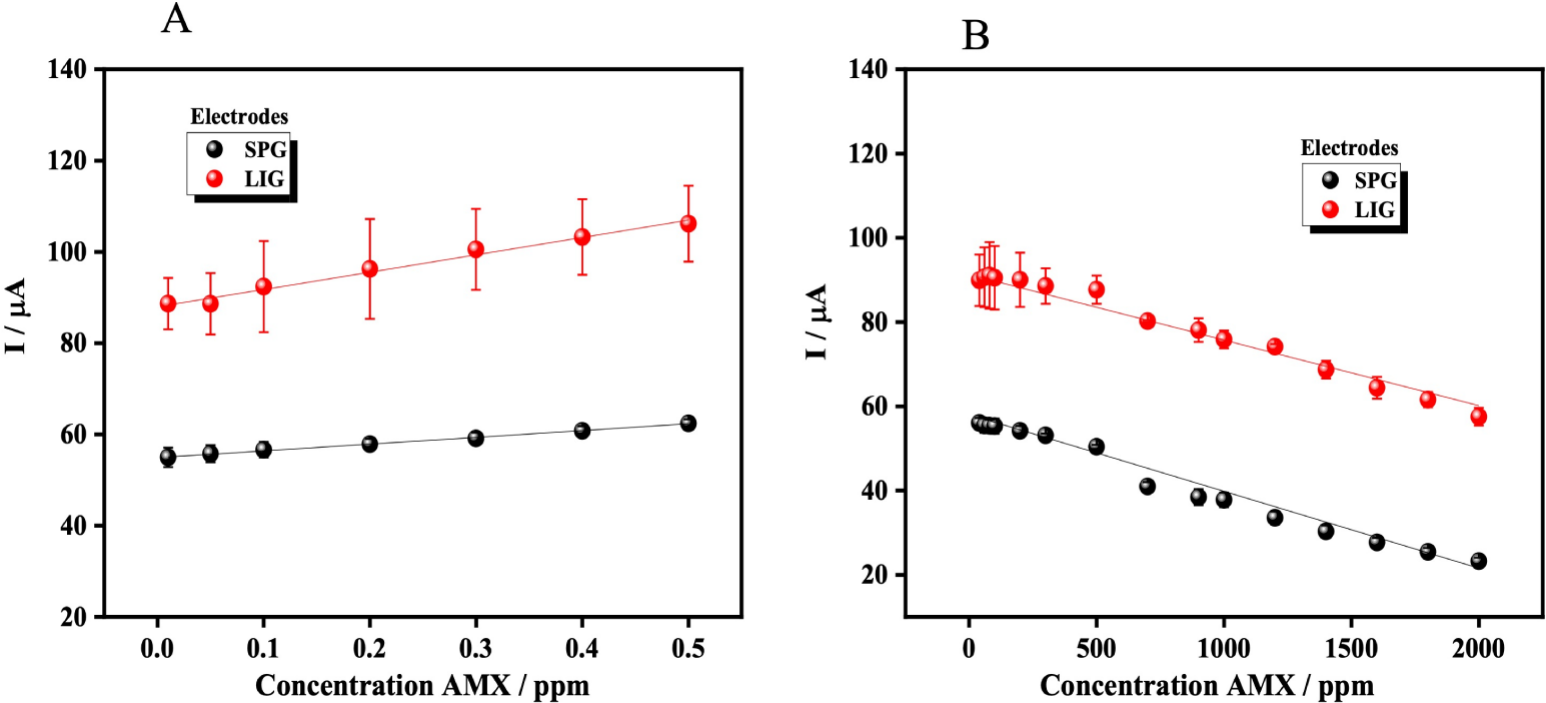
A) Comparison of AMX calibration curve in the concentration range from 0.01 to 0.5 ppm between LIG and SPG electrodes. B) Comparison of AMX calibration curve in the concentration range from 40 to 2000 ppm between LIG and SPG electrodes. The curves were obtained by AC using electrochemical oxidation methodology.

Although the functional behavior was identical for both electrode types, differences in the absolute oxidation current (*i*_o_) were observed. As discussed in Section E.2, these variations correlate with the distinct morphological evolution and roughness factors determined before and after EF operation. While SPG electrodes initially present a more homogeneous surface, they undergo a substantially larger increase in roughness under EF conditions (∼22%), whereas LIG electrodes display only minor morphological variation (∼6.7%), reflecting greater structural stability.

Beyond roughness, additional intrinsic parameters – including real electroactive surface area, defect density, electrical conductivity, and heterogeneous electron-transfer kinetics – also contribute to differences in current magnitude. LIG electrodes, characterized by a high density of edge-plane defects generated during laser processing, inherently favor enhanced faradaic responses, whereas SPG electrodes reflect microstructural features associated with ink composition and controlled manufacturing. These effects are well documented for carbon-based materials and are consistent with prior studies highlighting the sensitivity of electrochemical performance to surface morphology and graphitic disorder.^54–57^ Importantly, these factors influence only the amplitude of the measured current and do not modify the underlying EF-mediated oxidation mechanism.

Figure 4A–B compares the AMX calibration curves obtained with LIG and SPG electrodes in both concentration regimes. For the SPG platform, the low-concentration range (0.01–0.5 ppm) followed the regression *I* (μA) = 14.82·C_AMX_ + 54.91 (*r* ^2^ = 0.997), while the high-concentration range (40–2000 ppm) was described by *I* (μA) = −0.018·C_AMX_ + 58.06 (*r* ^2^ = 0.981). The limit of detection calculated for SPG electrodes (LOD_SPG_ = 0.021 ppm), based on the standard deviation of the blank (*n* = 9) and the slope of the most sensitive region, was lower than that obtained with LIG. This improvement reflects the higher point-to-point reproducibility afforded by commercially fabricated electrodes, as shown by the consistently smaller standard deviations observed for SPG measurements. In contrast, the intrinsic structural variability associated with laser-induced graphene can introduce greater dispersion in absolute current values, despite preserving identical mechanistic trends.

Taken together, these results demonstrate that the EF-assisted sensing strategy is robust, reproducible, and independent of the electrode fabrication method. The preservation of mechanistic behavior across two distinct graphene-based platforms confirms the generality of the dual-regime EF response. The wide dynamic range spanning more than 5 orders of magnitude, combined with sub-ppm detection limits and high reproducibility, positions this methodology as a competitive alternative to conventional electrochemical sensors. Its rapid response, minimal sample preparation, and low operational cost make it a powerful and practical complement to chromatographic and electrophoretic techniques for real-time monitoring of total emerging contaminants in complex water matrices. However, it is limited to detecting total ECs and does not allow identification of individual contaminants.

### 3.4 Multianalyte Evaluation of ECs Using the Optimized EF-Assisted LIG Sensor

Figure 5 shows the calibration curves obtained on LIG electrodes for a representative set of emerging contaminants—amoxicillin (AMX), ibuprofen (IBF), diclofenac (DCF), glyphosate (GLY), and estradiol—across the concentration range of 50–500 ppb (μg L^–1^). All analytes showed a linear increase in current with increasing concentration, confirming that the EF-assisted chronoamperometric (CA) response is consistent across compounds with markedly different physicochemical properties.

**Figure 5:**
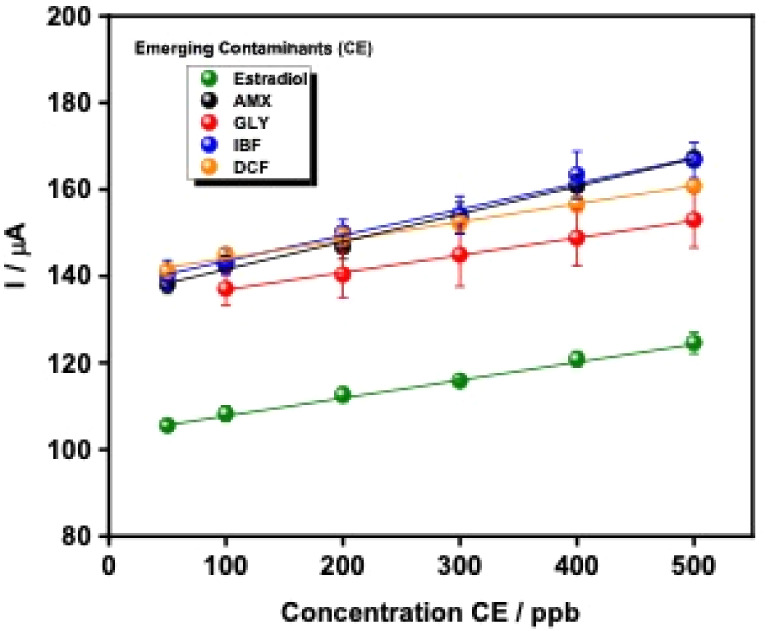
Calibration curves for pharmaceutical and herbicide CE determination in LIG electrodes, linear range of 50–500 ppb. The curves were obtained by CA using the electrochemical oxidation methodology.

For AMX, the regression equation was *I* (μA) = 0.0639·C + 135.16 (*r* ^2^ = 0.9974; *n* = 6 replicates per concentration), yielding a LOD = 0.085 ppb and a limit of quantification (LOQ) of 0.257 ppb, demonstrating high sensitivity at sub-ppb levels. DFC, IBF, and GLY showed calibration slopes of similar magnitude, with LODs of 0.142, 0.153, and 0.088 ppb, respectively. This similarity indicates that, under low-analyte conditions, ROS generated by the EF process remain in large excess, resulting in similar ROS-driven faradaic responses despite differences in molecular structure. In contrast, estradiol showed a slightly reduced current response, resulting in a higher LOD = 0.113 ppb. This behavior can be attributed to its lower diffusion coefficient due to increased hydrophobicity and molecular bulk, slower reaction kinetics with hydroxyl radicals compared to typical pharmaceutical compounds, and reduced oxidative susceptibility at neutral pH, where estradiol predominantly exists in its neutral form. These factors collectively limit mass transport and electron-transfer efficiency at the electrode surface, leading to a reduced electrochemical signal.^67–69^

Despite these differences in sensitivity, all analytes showed positive slopes, consistent with the mechanistic framework proposed in Figure 1C. Under optimized EF conditions, ROS generation remains sufficient to promote oxidative degradation of structurally diverse contaminants, yielding proportional increases in current. These results highlight the versatility of LIG electrodes and demonstrate that the EF-assisted CA methodology provides a general, rapid, and sensitive electrochemical platform for assessing emerging contaminants in complex aqueous matrices without the need for analyte-specific electrode modification or molecular recognition elements.

### 3.5 Extended Characterization of the Electrochemical Oxidation of AMX by HPLC-MS

To gain a deeper insight into the electrochemical oxidation mechanisms in solution and to substantiate the electro-Fenton-based degradation pathway proposed in this study, complementary validation was performed using high-performance liquid chromatography coupled with mass spectrometry (HPLC-MS). This approach not only verified the linearity of the developed electrochemical sensor but also confirmed the degradation products and their conversion pathways under different electrochemical conditions, allowing a robust characterization of the oxidation process. A full-scan mass spectrometric analysis was performed using a quadrupole time-of-flight mass spectrometer (QTOF-MS), acquiring data in both MS and MS/MS modes with a scan range of *m*/*z* 50–400 and a resolution of 30,000. A standard solution of AMX at 10 ppm in methanol was analyzed, and the extracted ion chromatogram (EIC) showed a retention time of 2.6 min for the intact AMX molecule (Figure S.3.A), confirming the purity of the standard solution.

In MS mode, the protonated molecular ion of AMX, [M + H]^+^ = 366.1118 *m*/*z*, was identified, as shown in Figure S.3.B. This signal corresponds to the molecular formula C_16_H_19_N_3_O_5_S, which is consistent with the previously reported fragmentation patterns. To further investigate the fragmentation pathway, an MS/MS experiment was performed in which *m*/*z* 366.100 was chosen as the precursor ion. Three predominant productions were detected: *m*/*z* 349.08, *m*/*z* 208.04, and *m*/*z* 114.00 (Figure S.3.C), corresponding to β-lactam ring cleavage and subsequent structural rearrangement, which is very consistent with the degradation pathways described in the literature.^70^ After characterizing AMX under nonoxidized conditions, a second set of analyzes was performed under the optimized electrochemical remediation conditions. These conditions included 400 ppb AMX in phosphate-buffered saline (PBS), potassium ferrocyanide (2.0 mmol L^–1^) and hydrogen peroxide (80 mmol L^–1^). Chromatographic analysis of the electrochemically treated AMX solution showed a significant shift in retention time to 4.6 min (Figure S.4.A), in contrast to the 2.6 min observed in methanol. This shift is attributed to changes in the polarity of the solvent, the formation of oxidation intermediates and interactions with other solution components.

Figure S.4.B–C show the corresponding mass spectra obtained before and after electrochemical oxidation. In MS mode, the characteristic AMX peak at *m*/*z* 366.127 is still observed; however, the MS/MS spectrum shows two major products at *m*/*z* 114.0371 and *m*/*z* 160.0426, which were subsequently used for AMX quantification. After electrochemical oxidation, two primary oxidation products were identified, confirming that AMX undergoes hydroxylation and structural changes upon oxidation. Prior to oxidation, the primary AMX precursor ion [M + H]^+^ = 366.11 *m*/*z* was detected with its characteristic MS/MS fragments (Figure 6A).

**Figure 6:**
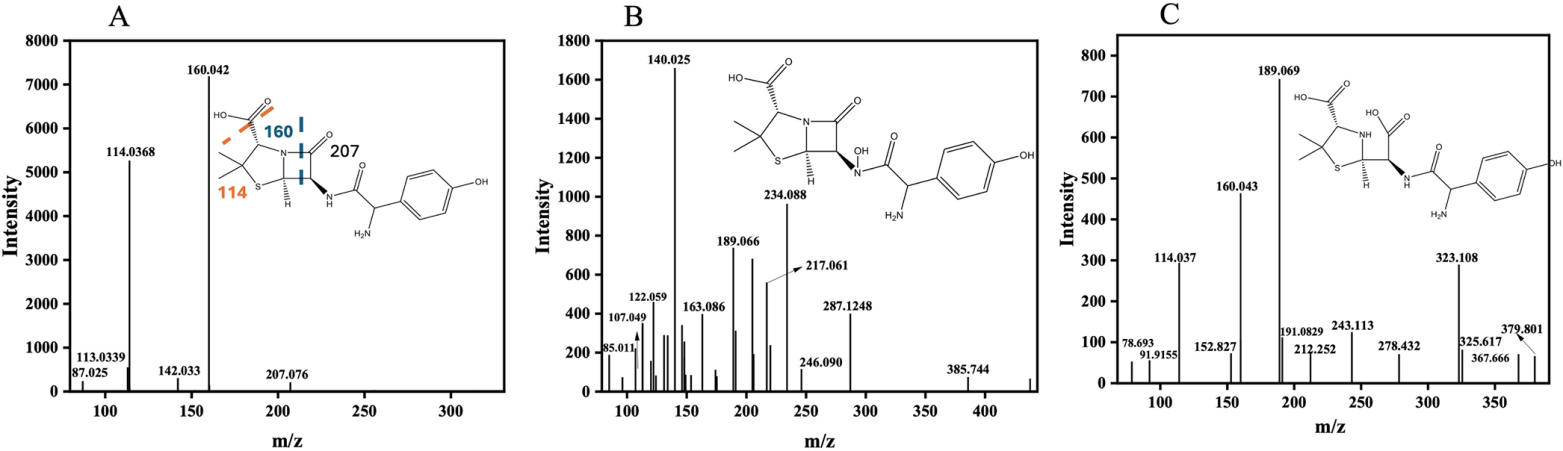
A) HPLC-ESI-TOF-MS/MS analysis of AMX and its fragmented degradation products, obtained in the electrochemical oxidation. A) AMX C_16_H_19_N_3_O_5_S, [M + H]^+^ = 366.11 *m/z*. B) Hydroxylation of AMX (C_16_H_19_N_3_O_6_S, [M + H]^+^= 382.10 *m*/*z*. C) Penicilloic acid of amoxicillin (C_16_H_21_N_3_O_6_S, [M + H]^+^ = 384.12 *m*/*z*).

After applying the electrochemical oxidation process, the first observed transformation was hydroxylation, which yielded a hydroxylated AMX derivative ([M + H]^+^ = 382.10 *m*/*z*) (Figure 6B), consistent with initial ROS attack on electron-rich sites of the molecule. The major fragments detected were *m*/*z* 234.088, 287.125, 189.066, and 140.025, indicating a sequential loss of ammonia (NH_3_) and carbon monoxide (CO), consistent with sequential loss of NH_3_ and CO, in agreement with previously reported AMX degradation pathways.^71^ Further oxidation led to β-lactam ring cleavage yielding amoxicillin-penicilloic acid ([M + H]^+^ = 384.12 *m*/*z*), confirmed by an accurate mass measurement with a deviation of 4.67 ppm. A diagnostic fragment at *m*/*z* 323.108 was attributed to decarboxylation, while the ion at *m*/*z* 189.0961 further confirmed the structural identity of the penicilloic-type product. The retention times, exact calculated masses, mass errors (ppm), and empirical formula assignments for all major protonated species and fragment ions are summarized in Table S.1 (Supporting Information). Collectively, these data indicate a progressive oxidation sequence involving initial hydroxylation, followed by β-lactam opening and downstream fragmentation under EF conditions.

Based on these results, a mechanistic degradation pathway for AMX under electrochemical oxidation conditions was proposed (Figure 7). In this framework, •OH generated through Fe^2+^/Fe^3+^ cycling in the presence of H_2_O_2_ initiates selective hydrogen abstraction or radical addition events, leading to hydroxylated intermediates. Subsequent oxidative steps promote β-lactam ring destabilization and conversion into penicilloic-type derivatives. Importantly, the relative preference for formal hydrogen transfer (FHT) versus radical adduct formation (RAF), as well as the susceptibility of specific atomic sites to ROS attack, cannot be inferred solely from MS data. Therefore, DFT calculations were carried out to quantify oxidation potentials, evaluate SET feasibility, and compute radical Fukui indices for the relevant AMX microspecies. These theoretical results, discussed in Section 3.6, provide a molecular-level interpretation of the experimentally observed transformation pattern and explain the predominance of specific oxidative pathways under EF conditions.

**Figure 7:**
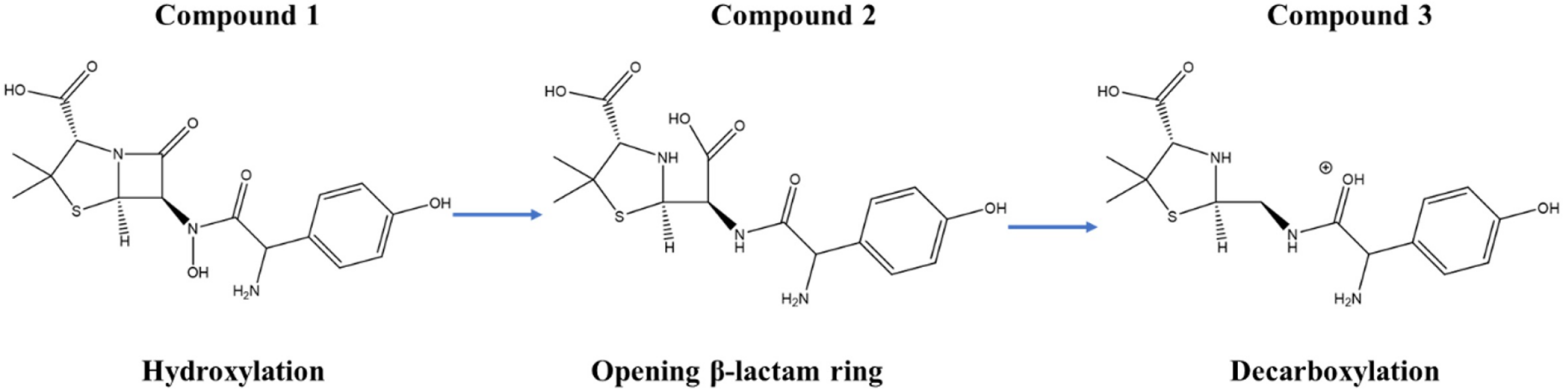
Possible degradation pathway of AMX.

To independently validate the electrochemical quantification of AMX and assess the efficiency of its electrochemical degradation, a calibration curve was constructed using HPLC-MS under optimized chromatographic conditions. The resulting regression equation, Area = 1.925·C_AMX_ + 29.3012 (*r* ^2^ = 0.998; *n* = 6 replicates per concentration), shown in Figure 8A, demonstrates excellent linearity over the tested concentration range and confirms the quantitative reliability of the HPLC-MS method. The electrochemical remediation of AMX was then evaluated as a function of oxidation time (200, 400, and 600 s) at a constant potential of 0.80 V. The corresponding HPLC-MS chromatograms (Figure 8B) show a clear and progressive reduction in the AMX peak area, indicating substantial degradation. The initial concentration of 524 ppb decreased to 392 ppb after 200 s, 243 ppb after 400 s, and 24.08 ppb after 600 s, yielding a final remediation efficiency of 95.4% (Figure 8C). These results confirm that the EF-assisted electrochemical process not only detects AMX but also effectively degrades it, supporting the dual sensing–remediation functionality of the platform.

**Figure 8:**
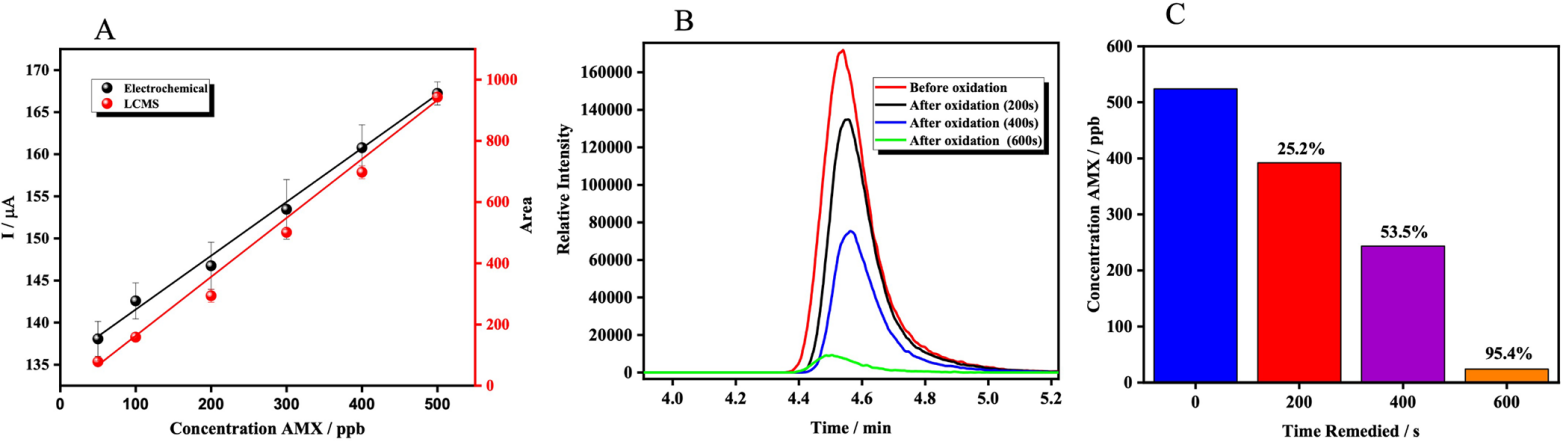
A) Calibration curve for AMX by HPLC-MS. B) Chromatogram of AMX before and after oxidation at different times. C) % AMX remediation at different oxidation times.

Both quantification approaches—CA and HPLC-MS—exhibited excellent linearity (*r* ^2^ > 0.99), demonstrating the robustness of the two independent methods. The EF-based electrochemical technique achieved a LOD of 0.085 ppb within the 50–500 ppb calibration range, while the HPLC-MS method achieved a slightly lower LOD of 0.073 ppb, confirming the high sensitivity of both approaches. All concentration points in both methods were determined in triplicate, ensuring precision and reproducibility. A comparative evaluation of the two methods highlights their complementary strengths. HPLC-MS provides unmatched selectivity, enabling identification of AMX and its degradation intermediates with molecular resolution. However, its high cost, need for specialized infrastructure, and extensive sample preparation limit its suitability for routine environmental monitoring. In contrast, the EF-assisted electrochemical method offers major practical advantages, including portability, low operational cost, minimal sample preparation, and real-time monitoring capability, making it highly suitable for decentralized water quality analysis.

Table 1 summarizes the analytical metrics and performance characteristics of the proposed graphene-based sensor relative to established methods for detecting and remediating emerging contaminants. In addition to achieving competitive or superior LOD and LOQ values, the EF-assisted LIG sensor stands out for its ability to simultaneously detect and degrade contaminants with a simple, rapid, economical, and field-deployable methodology.

**Table 1:** Comparison of the Relevant Parameters in This Work with the Reported CE Detection

EC	Detection range	LOD (ppb)	Detection technique	Reference
Ibuprofen	10–1000 mmol L^–1^	391.9	Cyclic voltammetry using a glassy carbon electrode.	^72^
Ibuprofen	2–10 ppm	100	Square wave voltammetry (SWV), using SPG electrodes.	^73^
Diclofenac	5.86–66.6 μ mol L^–1^	8.29	Screen-printed carbon electrode was modified with graphene/gadolinium(III) oxide (Gd_2_O_3_). Pulse Voltammetry was used to study the oxidation of Diclofenac.	^74^
Glyphosate	1.8 × 10 ^–5^ to 1.2 × 10 ^–3^ mol L^–1^	2.87	Graphite oxide paste electrode (GrO-PE) for the direct determination of GLY traces in groundwater samples, using SWV	^75^
Estradiol	0.1–1.0 μmol L^–1^	1.44	Using an electrode based on reduced graphene oxide and a metal complex porphyrin, was analyzed by differential pulse voltammetry in the determination of 17β-estradiol in a river water sample	^76^
AMX	0.195–14.6 μmol L^–1^	32.15	Determination of AMX in the presence of copper ions Cu (II) at carbon paste electrode. Using CA.	^77^
AMX	2.0–18.8 μmol L^–1^	43.85	Glassy carbon (GC) substrate modified with carbon black (CB) immobilized within a dihexadecylphosphate (DHP), using square-wave voltammetry	^78^
AMX	50–500 ppb (ng/mL^–1^)	0.085	CA detection of ECs by the electrochemical catalytic oxidation of EC in the presence of hydrogen peroxide, using LIG and SPG electrode	This work
Diclofenac		0.142		
Ibuprofen		0.153		
Glyphosate		0.088		
Estradiol		0.113		

### 3.6 Computational Analysis of EF-Mediated Oxidation Pathways

Under optimized EF conditions, •OH and HO_2_• are the primary oxidizing radicals responsible for EC degradation, promoting oxidation through three principal pathways: single-electron transfer (SET), formal hydrogen transfer (FHT), and radical adduct formation (RAF).^79,80^ The relative contribution of each mechanism depends on both thermodynamic driving forces and kinetic accessibility, which can be quantitatively assessed by molecular simulations. To determine which pathways are viable under the experimental conditions, DFT calculations were performed. Because radical reactivity is highly sensitive to protonation state—particularly for multifunctional organic molecules capable of proton exchange with the surrounding medium—acid–base speciation was first evaluated using reported p*K*_a_ values (Figure S.6). At pH 7.0, AMX exists predominantly as two microspecies: a zwitterionic form (molar fraction *x* = 0.76, net charge *q* = 0) and a monoanionic form (*x* = 0.24, *q* = −1), while the remaining emerging contaminants are dominated by a single protonation state under these conditions (Table 2). These dominant microspecies were subsequently used in all redox and radical reactivity calculations to ensure chemically consistent modeling aligned with the experimental EF environment.

**Table 2:** Molar Fraction (*x*), Electronic Charge (*q*, e^–^), Calculated Formal Reduction Potentials of 1 *e* ^–^ ( $E_{\mathrm{calc}}^{{}^{\circ}\prime }$ , V vs Ag/AgCl Sat.), and Symbols of Emergent Contaminants

EC	Symbol	*q*	*x*	$E_{\mathrm{calc}}^{{}^{\circ}\prime }$ ^a^
AMX	AMX^0^	0	0.76	1.35
	AMX^1–^	–1	0.24	1.51
DIC	DIC^1–^	–1	1.00	0.59
IBU	IBU^1–^	–1	0.99	1.49
EST	EST^1–^	0	1.00	0.97
GLY	GLY^2–^	–2	0.96	1.95

a Calculated at ωB97X-D/6–311+G­(2d,p) level with SMD­(H_2_O).

The computed formal one-electron oxidation potentials ( $E_{\mathrm{cal}}^{\prime }$ vs Ag/AgCl, saturated KCl, see eq S.1) show that, except for DIC (0.59 V), all ECs have oxidation thresholds ≥0.97 V, with AMX exceeding 1.3 V depending on its protonation state. These potentials are beyond the effective anodic window established experimentally (3.1–3.2), where no direct oxidation peaks were observed. Therefore, direct electron abstraction at the electrode surface is thermodynamically disfavored for AMX and most ECs. Instead, degradation proceeds via diffusion-controlled, ROS-mediated pathways, consistent with the radical steady-state model described by eq 10 and the EF kinetic framework developed in the preceding sections.

The formal redox potential of the •OH/OH^–^ couple is central to evaluating the thermodynamic viability of single-electron transfer (SET) pathways. Experimentally, this couple exhibits a high oxidation potential (∼1.70 V vs Ag/AgCl, saturated KCl),^81^ confirming the strong oxidizing character of •OH in aqueous systems. However, within our DFT/SMD framework, the calculated value for the •OH/OH^–^ couple is underestimated, reflecting the intrinsic difficulty of accurately modeling OH^–^ due to its highly localized charge density and strong specific solvation. This systematic offset must be considered when interpreting computed reaction energetics. The SET mechanism was modeled according to the general eq 11:

11 $$EC^{q}+•OH\to EC^{\left(q+1\right)•}+OH^{-}$$

Here, EC^*q*^ represents the dominant microspecies of the emerging contaminant at pH 7, and EC^(*q*+1)•^ denotes the corresponding radical product after one-electron oxidation. Reaction Gibbs free energies $\left({\Delta G}_{\mathrm{calc}}^{0}\right)$ for this process were computed for each EC (Table 3). The raw DFT results indicate that SET is exergonic for DIC^–^ and AMX^–^, while it is slightly endergonic for AMX^0^, IBU^–^, and GLY^2–^.

**Table 3:** Thermodynamic and Kinetic Parameters for Single-Electron Transfer (SET) Reactions between the Predominant Microspecies of ECs and •OH, Including Calculated and Semiempirical Gibbs Free Energies ( $\Delta G_{\mathrm{calc}}^{{}^{\circ}}$ , $\Delta G_{\mathrm{SE}}^{{}^{\circ}}$ ), Activation Free Energies ( $\Delta G_{\mathrm{calc}}^{‡}$ , $\Delta G_{\mathrm{SE}}^{‡}$ , kcal mol^–1^), and Bimolecular Rate Constants (*k*_calc_, *k*_SE_, M^–1^ s^–1^), Computed at the ωB97X-D/6–311+G­(2d,p) Level with SMD (H_2_O)^a^

EC	$\Delta G_{\mathrm{calc}}^{{}^{\circ}}$	$\Delta G_{\mathrm{calc}}^{\neq }$	*k*_calc_	$\Delta G_{\mathrm{SE}}^{{}^{\circ}}$	$\Delta G_{\mathrm{SE}}^{\neq }$	*k*_SE_
AMX^0^	2.91	24.5	5.0 × 10^–6^	–4.31	22.7	1.0 × 10^–4^
AMX^1–^	–0.82	23.1	1.8 × 10^–5^	–8.01	21.4	2.9 × 10^–4^
DIC	–18.5	14.2	2.3 × 10^2^	–25.7	13.2	1.4 × 10^3^
IBU	2.34	22.4	2.3 × 10^–4^	–4.88	20.7	4.5 × 10^–3^
EST	–9.51	18.0	3.8 × 10^–1^	–16.7	16.7	3.6 × 10^0^
GLY	12.88	33.8	1.0 × 10^–12^	5.66	31.7	3.7 × 10^–11^

a Details for these calculations are included in the Supporting Information.

However, accurate modeling of the •OH/OH^–^ redox couple is intrinsically challenging because OH^–^ is a small, negatively charged molecule with high charge density and strong specific hydration effects, readily forming hydrogen bonds that are not fully captured by continuum solvation models.^82^ Within our DFT/SMD framework, the $E_{cal}^{\prime }$ for •OH/OH^–^ is underestimated by approximately 0.31 V relative to the experimental value (∼1.70 V vs Ag/AgCl), indicating that the oxidizing strength of •OH is conservatively described in the computed thermochemistry. Consequently, slightly positive ${\Delta G}_{\mathrm{calc}}^{0}$ values should be interpreted with caution. After correction using the experimental •OH/OH^–^ potential (semiempirical ${\Delta G}_{SE}^{0}$ values in Table 3), SET becomes thermodynamically accessible for all Ecs except GLY, supporting the plausibility of electron-transfer contributions under EF conditions.

Therefore, to obtain more realistic thermodynamic estimates, we recalculated the SET reaction free energies using the experimental •OH/OH^–^ potential (semiempirical ${\Delta G}_{SE}^{0}$ values in Table 3) together with the calculated formal potentials of the ECs. After this correction, SET becomes thermodynamically accessible for all ECs except GLY, supporting the plausibility of an electron-transfer pathway under EF conditions. However, Marcus theory analysis of the corresponding activation free energies ( $\Delta G_{\mathrm{calc}}^{‡}$ and $\Delta G_{SE}^{‡}$ ),^82^ computed from DFT-derived nonadiabatic energy gaps, reveals relatively high kinetic barriers for AMX, IBU, and GLY. This results in bimolecular rate constants (*k*_calc_ and *k*_SE_, Table 3) well below diffusion-controlled limits, while only DIC exhibits barriers compatible with near-adiabatic, rapid SET. Thus, although electron transfer is thermodynamically accessible, it is unlikely to dominate AMX degradation under the EF conditions employed.

Given this limited kinetic favorability, alternative radical pathways were evaluated. Condensed Fukui indices (*f* ^0^)^18^ and Hirshfeld charge analyses^19^ were calculated to identify the most susceptible reactive centers for FHT and RAF mechanisms (Figure 9). The highest radical susceptibility values are localized at the sulfur atom of the thiazolidine ring and at aromatic positions of the phenolic moiety. Notably, hydrogen atoms do not rank among the most reactive sites, rendering FHT mechanistically disfavored. Instead, the electronic descriptors consistently indicate radical adduct formation (RAF) as the predominant pathway.

**Figure 9:**
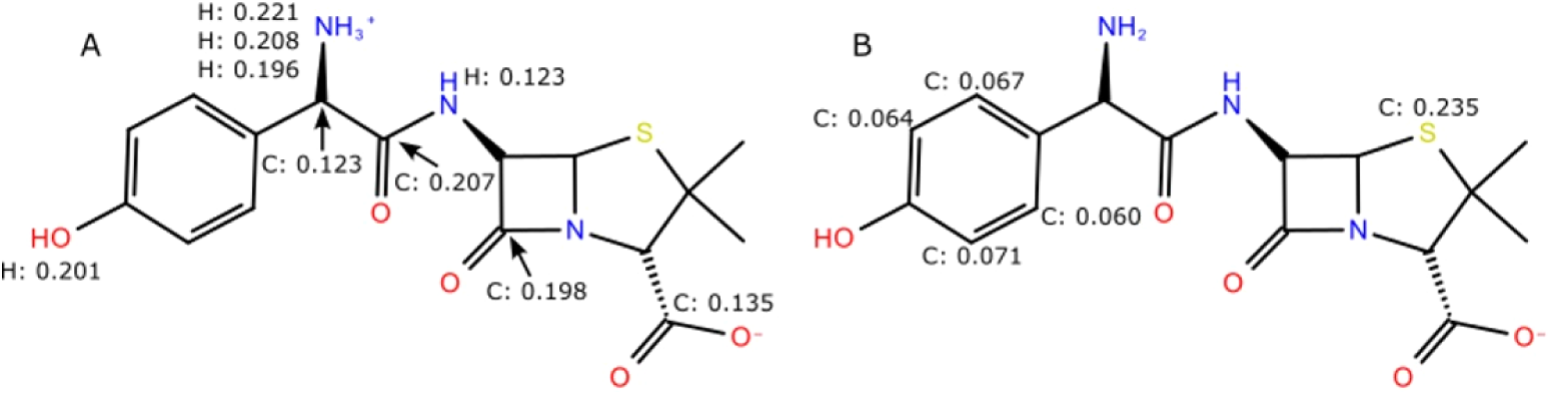
A) Partial atomic charges of the 10 most positively charged atoms in AMX^0^, obtained from Hirshfeld population analysis.^19^ B) Top five condensed Fukui indices^18^ for radical reaction of the AMX^–^ microspecies.

Complementary condensed Fukui indices and Hirshfeld-derived partial atomic charges for AMX^0^ and AMX^–^ (Figure S6, Supporting Information) reveal pronounced electrophilic character at the carbonyl carbons of the β-lactam ring and adjacent amide groups, along with high radical susceptibility localized at the sulfur atom of the thiazolidine ring and at aromatic positions of the phenolic moiety. This electronic distribution provides a molecular-level rationale for the experimentally observed β-lactam ring opening and formation of penicilloic-type intermediates (Section 3.5), as well as for the hydroxylated products identified by LC-MS/MS (Figure 6). Collectively, the DFT results indicate that AMX oxidation under EF conditions proceeds predominantly through radical adduct formation (RAF), followed by structural rearrangement and ring cleavage, while single-electron transfer plays at most a secondary role. The agreement among computed reactive centers, ROS kinetic considerations (eq 10), and experimentally detected transformation products establishes a unified mechanistic framework in which the electrochemical signal arises from ROS-mediated oxidation rather than direct anodic electron abstraction.^83^

### 3.7 Analysis of Real Samples Using the EF-Assisted Electrochemical Sensor

To assess the applicability of the proposed methodology in complex matrices, real wastewater samples subjected to different treatment processes were analyzed, including raw wastewater, microalgae-treated water, and water treated sequentially with microalgae and electro-Fenton. Targeted HPLC-MS/MS analysis quantified only a limited set of known pharmaceuticals (Supporting Table S.2), yielding total concentrations of 0.1923 ppb for raw water, 0.0192 ppb after microalgal treatment, and 0.0055 ppb after microalgae plus EF. However, numerous peaks present in the untreated wastewater chromatograms were not quantified (not shown), and the treated samples exhibited multiple new peaks absent in the raw matrix, consistent with transformation products formed during biological degradation and EF oxidation. These compounds could not be identified or quantified due to the lack of reference fragmentation patterns, as analytical standards required to construct calibration curves for the wide variety of oxidation products generated during advanced oxidation processes were unavailable. This limitation is well recognized: targeted chromatographic methods quantify only analytes for which standards or library spectra exist, systematically underestimating the total contaminant burden when unknown intermediates persist in the matrix. Similar behavior has been widely documented in advanced oxidation and biological remediation systems; for example, Ravelo-Nieto et al. (2023) demonstrated that early decolorization in heterogeneous Fenton treatment occurs despite the persistence of multiple intermediate species that remain invisible to conventional chromatographic workflows and can only be elucidated through high-resolution methods such as MALDI-TOF, but not quantified.

In contrast, the electrochemical methodology developed here quantifies the total concentration of electroactive and ROS-reactive emerging contaminants (total ECs) regardless of chemical identity. Because the EF mechanism oxidizes most organic molecules to common mineralization products (CO_2_, H_2_O, and, when applicable, NO_*x*_/SO_*x*_ derivatives), the resulting CA signal reflects the overall oxidative load of the sample. Eight replicates per condition yielded average signals of 43.67 ± 0.68 μA for raw wastewater, 33.61 ± 2.78 μA for microalgae-treated water, and 37.35 ± 4.67 μA for microalgae plus EF, corresponding to total EC concentrations of 0.69, 0.23, and 0.25 ppb, respectively. Although both treatment processes decreased the overall contaminant load, the load of emerging contaminants measured by the electrochemical method was higher than that inferred from HPLC-MS/MS. This indicates that chromatographic quantification underestimates the true concentration of emerging contaminants, as it captures only identifiable parent molecules, whereas the electrochemical approach accounts for the entire pool of ROS-oxidizable species, including transformation products from microalgal and EF treatment. Thus, the electrochemical method provides a more comprehensive representation of the total oxidative burden of the sample. The electrochemical measurements do not indicate overestimation but instead reflect their intrinsic ability to capture the full ensemble of ROS-reactive contaminants—an analytical capability fundamentally inaccessible to targeted chromatography.

This distinction has important implications for environmental monitoring. While HPLC-MS/MS remains indispensable for structural identification and mechanistic studies, it cannot provide an exhaustive assessment of treatment performance in systems that generate unidentified transformation products. The EF-assisted LIG/SPG sensor, by contrast, delivers a holistic, treatment-agnostic, and matrix-inclusive measure of total EC burden. Overall, these results demonstrate that the proposed electrochemical platform constitutes a robust, sensitive, and realistic approach for evaluating water quality in complex matrices, overcoming the intrinsic blind spots of chromatographic techniques and providing a valuable tool for validating remediation processes and supporting environmental risk assessment.

## 4 Conclusions

This study establishes portable electrochemical sensors based on laser-induced graphene (LIG) and screen-printed graphene (SPG) electrodes as sensitive, robust, and versatile platforms for assessing total emerging contaminants (ECs) in water. Under optimized electro-Fenton (EF) conditions, the system achieved sub-ppb detection limits for amoxicillin (AMX) (LOD = 0.085 ppb) with excellent linearity (*r* ^2^ > 0.99) and strong agreement with high-resolution HPLC-MS, confirming quantitative reliability across diverse aqueous matrices. Importantly, the EF-assisted electrochemical response reflects the cumulative burden of oxidizable organic species rather than only individually targeted analytes. Because the EF process converts a wide range of organic contaminants into reactive intermediates and partial mineralization products, the measured current integrates the total ROS-reactive pool, overcoming the intrinsic selectivity limitations of targeted chromatographic methods that rely on available analytical standards. LC-ESI-QTOF-MS/MS analysis demonstrated that EF treatment induces chemically meaningful AMX transformations, including hydroxylation and β-lactam ring opening leading to penicilloic-type intermediates. These findings confirm that the electrochemical signal originates from ROS-mediated oxidation rather than direct anodic electron transfer. Application to real wastewater samples further revealed that the electrochemical platform reports a broader oxidizable organic load than HPLC-MS/MS, capturing both parent compounds and transformation products generated during microalgal and EF treatments. Thus, the method provides a more realistic and integrated assessment of oxidative contaminant burden in complex environmental matrices.

The combined experimental and computational results provide molecular-level insight into the EF degradation mechanism. Density functional theory (DFT) calculations indicate that, under the applied conditions, single-electron transfer plays at most a secondary role, whereas radical adduct formation (RAF) dominates AMX oxidation. Fukui indices and Hirshfeld charge analyses identify preferential radical attack at the sulfur atom of the thiazolidine ring and aromatic sites of the phenolic moiety, while pronounced electrophilic character at the β-lactam carbonyl carbons rationalizes ring-opening reactions. These predictions are fully consistent with the transformation products identified by LC-MS/MS and integrate electrochemical ROS generation, calculated energetics, and spectrometric evidence into a unified mechanistic framework. Overall, the LIG-based EF sensor represents a rapid, low-cost, and field-deployable alternative to conventional analytical instrumentation. Its ability to quantify total oxidizable contaminant burden at ultratrace levels, combined with mechanistic interpretability and operational simplicity, positions this platform as a promising tool for decentralized water quality monitoring, treatment performance evaluation, and environmental risk assessment. This work lays the groundwork for extending graphene-based EF electrochemical strategies to a broader spectrum of emerging contaminants and complex environmental systems.

## Supplementary Material



## Data Availability

The data sets generated during and/or analyzed during the current study are available from the corresponding authors on request.

## Notes

Electrochemical characterization of SPG and LIG electrodes, including sensor configurations and cyclic voltammetry stability tests (Figures S.1–S.2); chromatographic and mass spectrometric analysis of AMX before and after electrochemical oxidation, including EIC, MS, and MS/MS spectra (Figures S.3–S.4); identification and structural assignment of AMX degradation products and intermediates by LC-TOF-MS/MS, including retention times, exact masses, and fragmentation patterns (Table S.1); spin density distributions of radical intermediates for AMX and other emerging contaminants (Figure S.5); p*K*_a_-based speciation diagrams of all studied contaminants under experimental conditions (Figure S.6); FE-SEM and EDS analysis of morphological and compositional evolution of SPG and LIG electrodes before and after electro-Fenton operation (Figure S.7); targeted HPLC-MS/MS quantification of selected pharmaceuticals in real wastewater samples before and after treatment (Table S.2); thermodynamic data for radical reactions and single-electron transfer processes in aqueous phase, including hydration effects (Tables S.3–S.4); detailed computational methodology, including calculation of oxidation potentials, Gibbs free energies, Marcus theory treatment for electron-transfer kinetics, Fukui index analysis, and cluster–continuum modeling of hydroxyl radicals (Section S.1) (PDF)
