# Supplementary material for: Graphene-Based Electrochemical Sensors for the Determination of Pharmaceutical- and Agricultural-Based Emerging Contaminants in Water
Source: Anal Chem. 2026 Apr 30;98(18):13409–24. doi: 10.1021/acs.analchem.5c07462 (PMC13177291; doi:10.1021/acs.analchem.5c07462)
Supplement: Supplementary file 1 [file ac5c07462_si_001.pdf]

Supplementary Information

Graphene-based Electrochemical Sensors for the Determination of Pharmaceutical- and Agricultural-based Emerging Contaminants in Water

Drochss Pettry Valencia[*,a], Gloria Crespo[b], Leonardo Muñoz-Rugeles[a], Gustavo Lara[b], Andres Jaramillo-Botero[b,c,**].

[a] *Escuela de Química, Universidad Industrial de Santander, Carrera 27, Calle 9 Ciudad Universitaria, AA 678, Bucaramanga, Santander, Colombia*
[b] *iOMICAS Research Institute, Pontificia Universidad Javeriana, Calle 17 # 121B-155, Cali, Valle del Cauca 760031, Colombia.*
[c] *Chemistry and Chemical Engineering, California Institute of Technology, Pasadena, CA, 91125, United States*

Corresponding authors: *drpetval@uis.edu.co, **ajaramil@caltech.edu

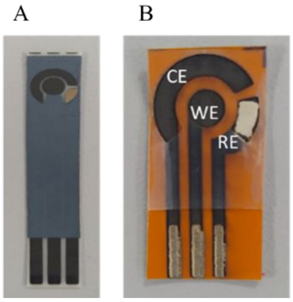

Figure S.1. *(A) SPG and (B) LIG sensor configurations*

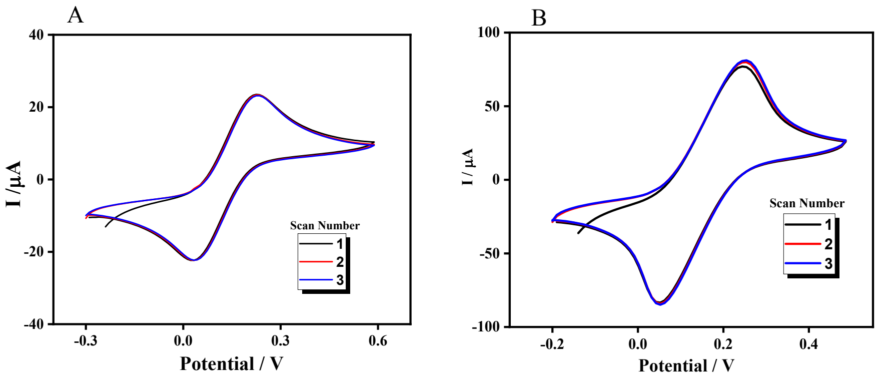

Figure S.2. *A) VC ferri/ferro 2.0 mmol L-1 at 50 mV/s in SPG. B). VC ferri/ferro 2.0 mmol L-1 at 50 mV/s in LIG.*

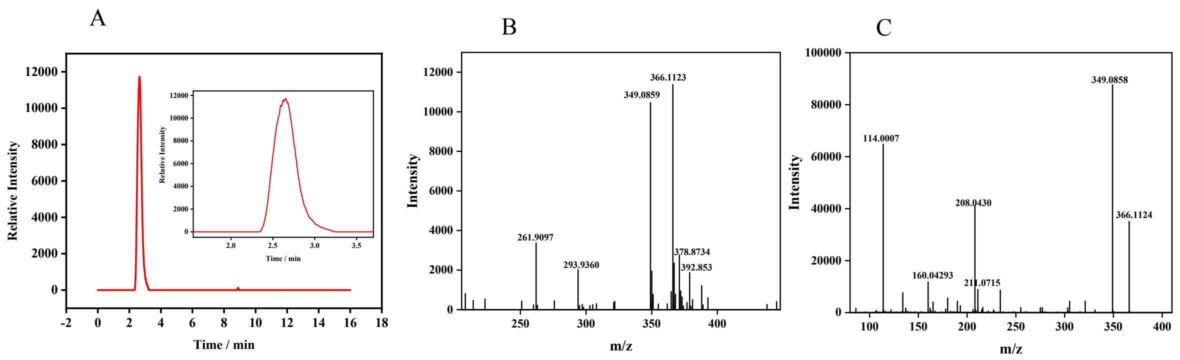

Figure S.3. *A) Chromatogram of AMX 10 ppm. B) Mass spectrometer of Amx 10 ppm. C) Mass spectrometer/mass spectrometer of Amx 10 ppm.*

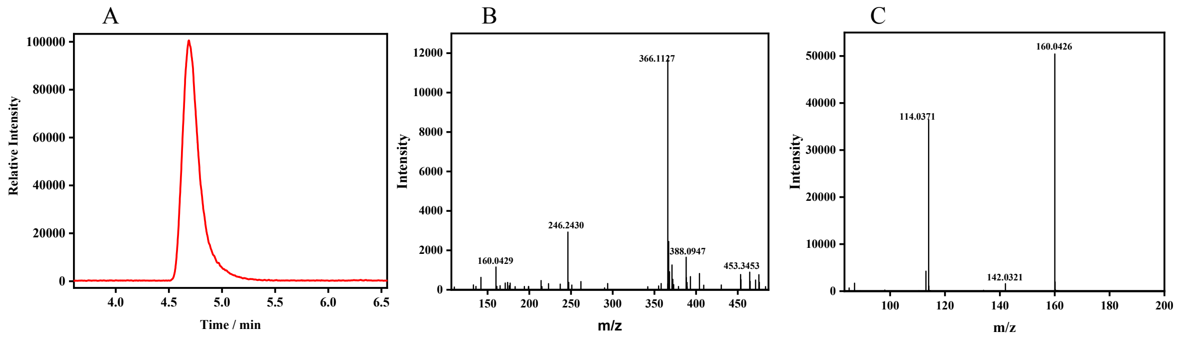

Figure S.4. *A) Chromatogram of AMX 400 ppb before and after electrochemical oxidation. B) Mass spectrometer of Amx 400 ppb. C) Mass spectrometer/mass spectrometer of Amx 400 ppb*

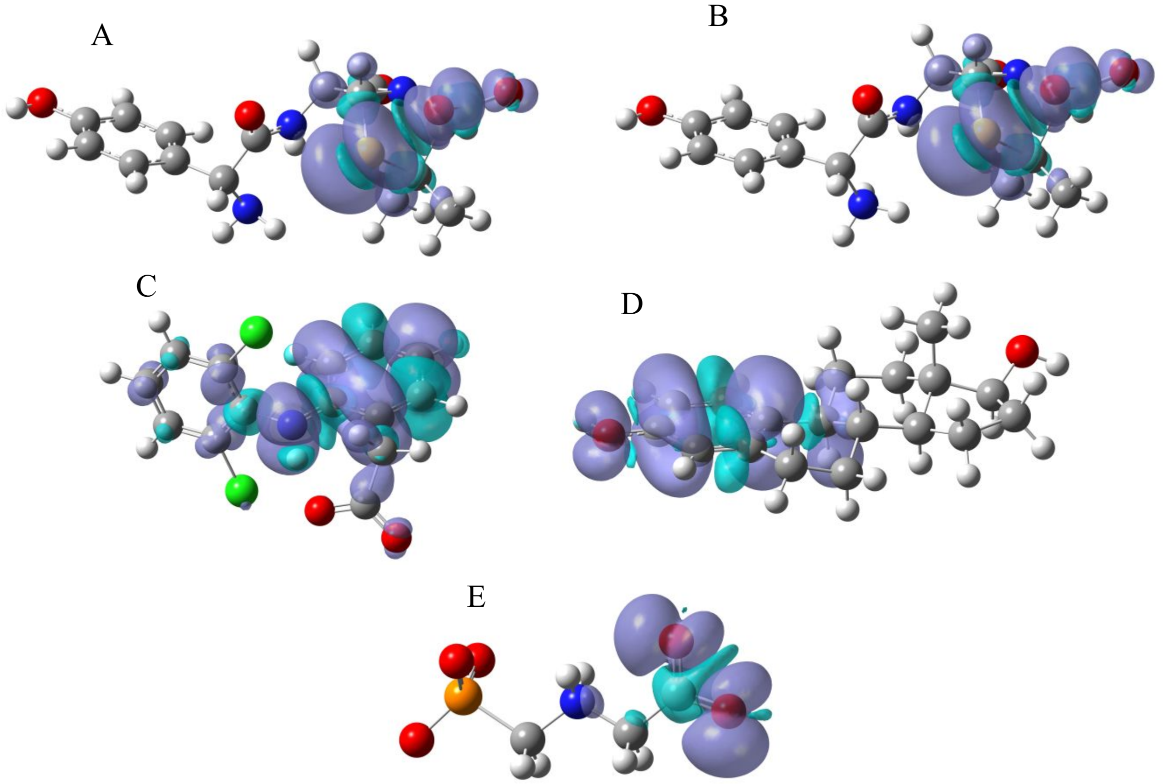

Figure S.5. *Spin density for free radical yielded by oxidation of (a) AMX^{1-}, (b) AMX^{0}, (c) DIC^{1-}, (d) IBU^{1-} and (e) GLY^{2-}.*

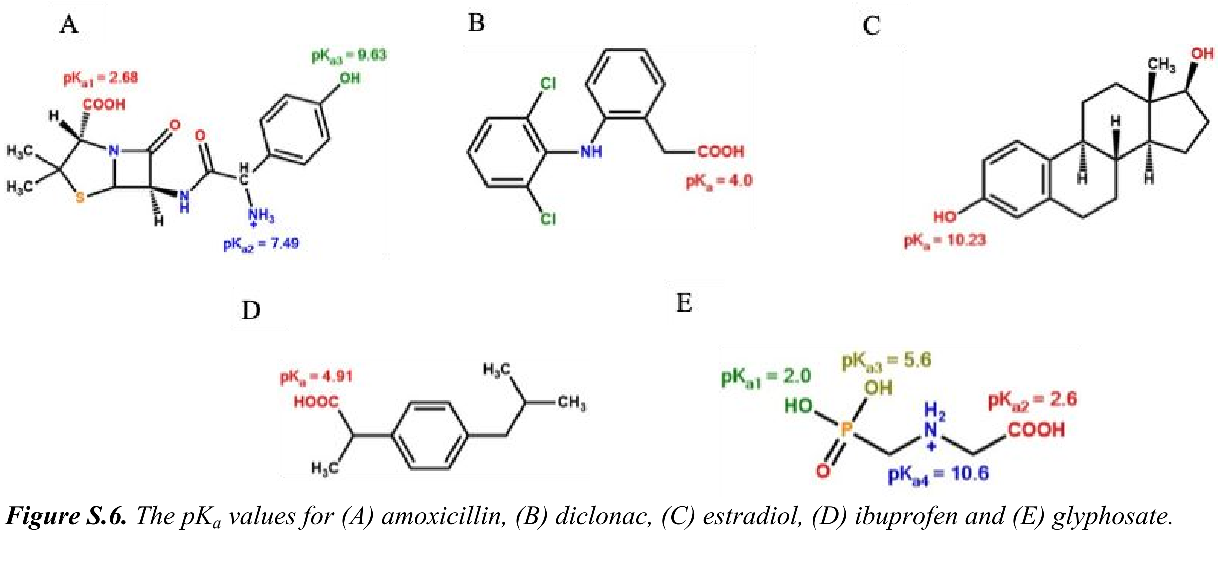

Figure S.6. *The pK_a values for (A) amoxicillin, (B) diclonac, (C) estradiol, (D) ibuprofen and (E) glyphosate.*

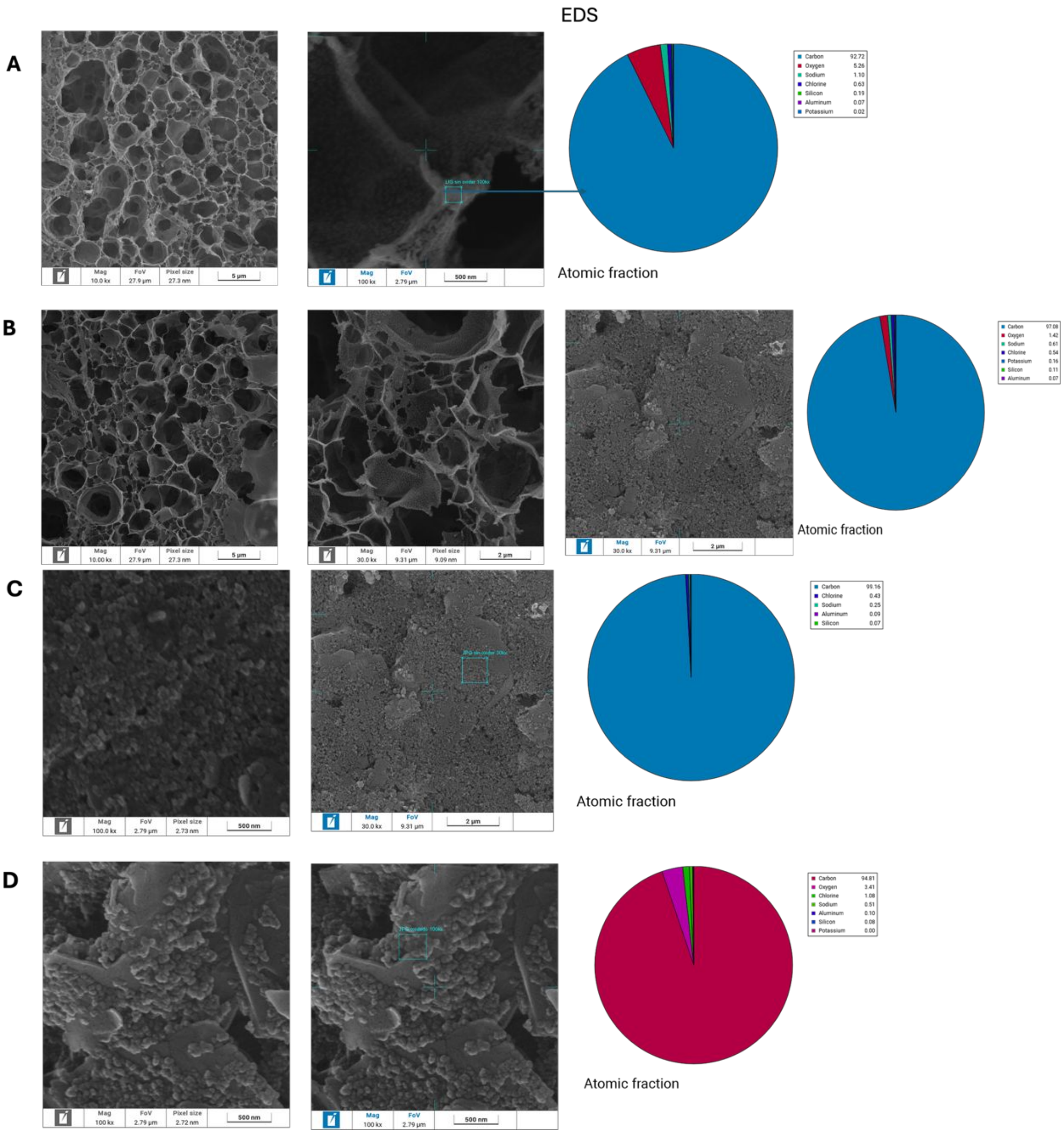

Figure S.7. *FE-SEM and EDS characterization of electrode surface evolution before and after the electro-Fenton (EF) process at 0.80 V vs Ag/AgCl during AMX oxidation. (A) Unoxidized LIG electrode; (B) LIG after EF oxidation; (C) Unoxidized SPG electrode; (D) SPG after EF oxidation.*

Table S.1. *Degradation products of AMX by LC-TOF-MS/MS and fragment ions by electrochemical oxidation process*

COMPOUND	RETENTION TIME (MIN)	ION FORMULA	CALCULATED MASS (M/Z)	ERROR (PPM)
**AMOXICILLIN**	**4.463**	**$C_{16}H_{19}N_3O_5S$**	**366.1114**	**0.273141**
		$C_{10}H_{10}N_2O_3$	207.0766	0.8716
		$C_6H_9NO_2S$	160.0424	-1.6983
		C_6H_7NOS	142.0327	4.1419
		C_5H_7NS	114.0368	-3.5086
		$C_4H_4N_2O_2$	113.0341	-3.9849
		C_4H_6S	87.025	-14.8936
**HYDROLYSED AMOXICILLIN**	**2.98**	**$C_{16}H_{19}$N3O6S**	**382.1064**	
		$C_{11}H_{11}N_3O_3$	234.0878	2.0565
		$C_{11}H_8N_2O_3$	217.061	1.0876
		$C_{10}H_8N_2O_2$	189.0662	1.7913
		$C_9H_8N_2O$	161.0706	-2.0917
		C_8H_7NO	134.0601	0.4779
		C_7H_6O	107.0496	4.2731
		$C_{10}H_{10}N_2O_2$	191.0818	1.5614
		$C_{10}H_7NO_2$	174.0539	-6.0951
		$C_8H_5NO_2$	148.0388	-3.3893
		C_9H_7NO	146.0605	3.1549
		$C_9H_{10}N_2O$	163.0862	-2.4067
		$C_8H_8N_2O$	149.0699	-6.8696
		C_9H_9NO	148.0748	-6.0225
		C_8H_6NO	133.0519	-2.406
		C_7H_7NO	122.0596	-3.6004
		$C_5H_5N_3O_2$	140.0453	-1.0676
		$C_4H_4N_2O_2$	113.0348	2.1573
		$C_3H_4N_2O$	85.038	-19.2524
		$C_6H_9NO_3S$	176.0364	-6.7209
		$C_5H_6O_2S$	131.0156	-4.0637
		C_5H_4OS	113.0049	-5.877
		C_3H_2OS	86.9906	7.9141
**AMOXICILLIN PENICILLINIC ACID**	**2.52**	**C $_{16}H_{21}N_3O_6S$**	**384.1223**	**-1.473489**
		$C_{15}H_{18}N_2O_4S$	323.1075	4.67
		$C_7H_{12}N_2O_2S$	189.0691	-0.6242
		$C_6H_9NO_2S$	160.0431	2.6874
		C_5H_7NS	114.0375	2.6465
		$C_{14}H_{14}N_2O_2$	243.1134	2.4646
		$C_{10}H_{10}N_2O_2$	191.083	7.79

Table S.2. *Targeted HPLC-MS/MS Quantification of Selected Pharmaceuticals in Real Wastewater Samples*

**Analyte**	**Raw Water (ppb)**	**Microalgae-Treated (ppb)**	**Microalgae + Electro-Fenton (ppb)**
Carbamazepine	0.01733	0.00092	0.00000
Diclofenac	0.00729	0.00380	0.00012
Gemfibrozil	0.00952	0.00141	0.00040
Ibuprofen	0.05057	0.00350	0.00000
Naproxen	0.10759	0.00963	0.00500
**Total (sum)**	**0.19230**	**0.01924**	**0.00552**

Table S.3. *Reaction free energy (ΔG°, kcal/mol) between the contaminants and the OH• radical solvated with explicit water molecules.*

**Contaminant**	**Fraction**	**Number of H_2O molecules**					
		**0**	**1**	**2**	**3**	**4**	**5**
AMX	AMX^0	13.6	8.27	3.87	3.39	3.44	2.91
	AMX^-	9.87	4.55	0.14	-0.34	-0.29	-0.82
DIC	DIC^-	-7.75	-13.1	-17.5	-18.0	-17.9	-18.5
IBU	IBU^-	13.3	7.71	3.30	2.82	2.87	2.34
EST	EST^0	1.18	-4.15	-8.55	-9.03	-8.98	-9.51
GLY	GLY^{2-}	23.6	18.2	13.8	13.4	13.4	12.9

Table S.4. *Reaction free energies (ΔG°_{calc}, kcal/mol) for single electron transfer between the contaminants and the OOH• radical.*

**Contaminant**	**SET reactions**	ΔG°_{calc}
AMX	$AMX^0 + OH^\bullet \rightarrow AMX^{+\bullet} + OH^-$	35.9
	$AMX^- + OH^\bullet \rightarrow AMX^\bullet + OH^-$	39.6
DIC	$DIC^- + OH^\bullet \rightarrow DIC^\bullet + OH^-$	18.3
IBU	$IBU^- + OH^\bullet \rightarrow IBU^\bullet + OH^-$	39.1
EST	$EST + OH^\bullet \rightarrow EST^{+\bullet} + OH^-$	27.2
GLY	$GLY^{2-} + OH^\bullet \rightarrow GLY^{-\bullet} + OH^-$	49.6

S.1. Formal Oxidation Potentials, Single Electron Transfer and Fukui indexes calculations

The oxidation potential of the emerging contaminants (ECs) were obtained from the direct oxidation method[1] [REF]. Calculations were performed for all relevant EC microspecies under the EF experimental conditions. Oxidation potentials ($E^{\circ\prime}_{calc}$) were calculated from the Nerst equation, as follows:

$$E^{\circ\prime}_{calc} = \frac{\Delta_r G^{\circ}_{O}}{n_e F} - E^{\circ}_{ABS} - E^{\circ}_{Ag/AgCl} \qquad (S.1),$$

where n_e is the number of electrons involved in the oxidation process ($n_e = 1$ for all the cases), F is the Faraday constant, E°_{ABS} is the absolute electrochemical reference potential used in quantum chemistry calculations of redox potentials ($E^{\circ}_{ABS} = 4.44\ V$) [2] [REF], $E^{\circ}_{Ag/AgCl}$ is the saturated Ag/AgCl pseudo-reference electrode potential used in the experimental section ($E^{\circ}_{Ag/AgCl} = 0.19\ V$ versus the standard hydrogen electrode), and $\Delta_r G^{\circ}_{O}$ is the reaction free energy for the oxidation of the reactive species, given by:

$$\Delta_r G^{\circ}_{O} = G^{+}_{sol}\left(g^{+}_{sol}\right) - G^{0}_{sol}\left(g^{0}_{sol}\right) \qquad (S.2)$$

$$G^{i}_{sol}\left(g^{i}_{sol}\right) = E^{i}_{sol}\left(g^{i}_{sol}\right) + G^{i}_{RRHO}\left(g^{i}_{sol},T\right) \qquad (S.3)$$

$G^{+}_{\mathrm{sol}}(g^{+}_{\mathrm{sol}})$ and $G^{0}_{\mathrm{sol}}(g^{0}_{\mathrm{sol}})$ denote the solution-phase Gibbs free energies of the oxidized and non-oxidized species, respectively, where $g$represents the optimized geometry of each species. The subscript "0" does not imply electrical neutrality, as the non-oxidized species may carry either a positive or negative charge. For the i-th species, whether oxidized or non-oxidized, $G^{i}_{\mathrm{sol}}(g^{i}_{\mathrm{sol}})$ is calculated from the corresponding internal energy $E^{i}_{\mathrm{sol}}(g^{i}_{\mathrm{sol}})$ and the thermodynamic contribution $G^{i}_{\mathrm{RRHO}}(g^{i}_{\mathrm{sol}},T)$, evaluated using the rigid-rotor harmonic-oscillator (RRHO) approximation at a temperature of 298.15 K [1] [REF]. Because the single-electron transfer (SET) reactions considered involve two solutes converting into two solutes ($\Delta n = 0$), no 1 atm → 1 M standard-state correction was required for ΔG°. To account for known limitations of continuum solvation in describing the •OH /OH⁻ redox couple, reaction free energies were additionally corrected using the corresponding experimental formal potential, yielding semiempirical reference values [3] [REF].

The single-electron transfer (SET), formal hydrogen transfer (FHT), and radical adduct formation (RAF) were explored to elucidate the reaction mechanism for ECs oxidation in the EF process in this study. Vertical electron-transfer energies (ΔE) were obtained from single-point calculations of the product electronic state at the optimized reactant geometry using the non-equilibrium SMD formalism, in which the slow solvent polarization is frozen while the fast electronic component relaxes. These values were used to evaluate the reorganization energy (λ, equation 2), and activation free energies ($\Delta G^{\ddagger}$) were estimated using Marcus theory, (equation 3) [4] [REF]. Bimolecular rate constants were subsequently computed using the Eyring equation for second-order reactions in solution, yielding rate constants in $M^{-1}s^{-1}$ (equation 4) [5] [REF] with $C^{\circ} = 1\ mol\ L^{-1}$:

$$\Delta G^{\ddagger} = \frac{\lambda}{4}\left(1 + \frac{\Delta G^{\circ}}{\lambda}\right)^2 \quad (2)$$

$$\lambda = \Delta E - \Delta G^{\circ} \quad (3)$$

$$k = \frac{k_B T}{h}\frac{1}{C^{\circ}} e^{-\frac{\Delta G^{\ddagger}}{RT}} \quad (3)$$

The hydroxyl radical (•OH) was modeled using a cluster–continuum approach, including explicit hydration with 3–5 water molecules. Spin-density distributions of the resulting radical intermediates were analyzed to assess charge and spin delocalization following electron transfer. The hydroxyl radical (•OH) was modeled using a cluster–continuum approach, including explicit hydration with 3–5 water molecules. Spin-density distributions of the resulting radical intermediates were analyzed to assess charge and spin delocalization following electron transfer.

Condensed Fukui indexes for radical reaction (f^0) [6][REF] of AMX were calculated according to:

$$f^0 = \frac{1}{2}\left[P_A(N-1) - P_A(N+1)\right],$$

$$\sum f^0 = 1;$$

where P_A denotes the atomic population of atom A in a given AMX microspecies. The terms $N-1$ and $N+1$ indicate that population analysis was performed for the reduced and oxidized forms of the AMX microspecies. Accordingly, condensed Fukui indices were obtained by performing calculations on the AMX^0 zwitterion and AMX^- anion in its reduced and oxidized forms.

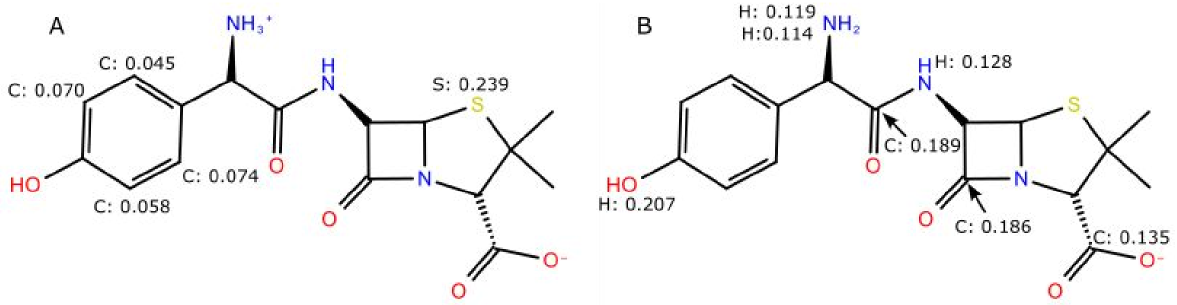

Figure S6. A) Top five condensed Fukui indices (f^0) for radical reaction of the AMX^0 microspecies. (B) Partial atomic charges of the ten most positively charged atoms in AMX^-, obtained from Hirshfeld population analysis.

1. References

1. Belić, J., Förster, A., Menzel, J. P., Buda, F. & Visscher, L. Automated assessment of redox potentials for dyes in dye-sensitized photoelectrochemical cells. *Physical Chemistry Chemical Physics* **24**, 197–210 (2022).
2. Trasatti, S. The absolute electrode potential: an explanatory note (Recommendations 1986). **58**, 955–966 (1986).
3. Eyring, H. The Activated Complex in Chemical Reactions. *J. Chem. Phys.* **3**, 107–115 (1935).

4. Marenich, A. V, Cramer, C. J. & Truhlar, D. G. Universal Solvation Model Based on Solute Electron Density and on a Continuum Model of the Solvent Defined by the Bulk Dielectric Constant and Atomic Surface Tensions. *J. Phys. Chem. B* **113**, 6378–6396 (2009).
5. Marcus, R. A. Chemical and electrochemical electron-transfer theory. *Annu. Rev. Phys. Chem.* **15**, 155–196 (1964).
6. Yang, Weitao. & Mortier, W. J. The use of global and local molecular parameters for the analysis of the gas-phase basicity of amines. *J. Am. Chem. Soc.* **108**, 5708–5711 (1986).
